# Clean-Room Lithographical Processes for the Fabrication of Graphene Biosensors

**DOI:** 10.3390/ma13245728

**Published:** 2020-12-15

**Authors:** Patrícia D. Cabral, Telma Domingues, George Machado, Alexandre Chicharo, Fátima Cerqueira, Elisabete Fernandes, Emília Athayde, Pedro Alpuim, Jérôme Borme

**Affiliations:** 1 International Iberian Nanotechnology Laboratory, 4715-330 Braga, Portugal; patricia.silva@inl.int (P.D.C.); telma.domingues@inl.int (T.D.); george.junior@inl.int (G.M.J.); alexandre.chicharo@inl.int (A.C.); fatima.cerqueira@inl.int (F.C.); elisabete.fernandes@inl.int (E.F.); jerome.borme@inl.int (J.B.); 2 Center of Physics, University of Minho, 4710-057 Braga, Portugal; 3 Center of Mathematics, University of Minho, 4710-057 Braga, Portugal; mefqa@math.uminho.pt

**Keywords:** graphene, microfabrication, biosensors, dielectric passivation, clean surfaces

## Abstract

This work is on developing clean-room processes for the fabrication of electrolyte-gate graphene field-effect transistors at the wafer scale for biosensing applications. Our fabrication process overcomes two main issues: removing surface residues after graphene patterning and the dielectric passivation of metallic contacts. A graphene residue-free transfer process is achieved by using a pre-transfer, sacrificial metallic mask that protects the entire wafer except the areas around the channel, source, and drain, onto which the graphene film is transferred and later patterned. After the dissolution of the mask, clean gate electrodes are obtained. The multilayer SiO_2_/SiN_x_ dielectric passivation takes advantage of the excellent adhesion of SiO_2_ to graphene and the substrate materials and the superior impermeability of SiN_x_. It hinders native nucleation centers and breaks the propagation of defects through the layers, protecting from prolonged exposition to all common solvents found in biochemistry work, contrary to commonly used polymeric passivation. Since wet etch does not allow the required level of control over the lithographic process, a reactive ion etching process using a sacrificial metallic stopping layer is developed and used for patterning the passivation layer. The process achieves devices with high reproducibility at the wafer scale.

## 1. Introduction

Since first obtained by the exfoliation of graphite [1], graphene has been explored in numerous applications [2,3,4] due to its outstanding properties, such as high carrier mobility [2,4], low intrinsic electronic noise [4,5], chemical stability [4,5,6], high sensitivity to electric charges in its vicinity [2,4], and record surface-to-volume ratio [2,5]. Graphene has been used as the building block for various electronic biosensing systems, with high sensitivity [3]. Among them, electrochemical sensors [7] and field-effect transistors (FETs) are the most common. Graphene FETs (GFETs) make use of graphene sensitivity to electric fields and charges [4,8] to produce the output signal of the sensor, operating at low voltages [8]. Additionally, GFETs are compatible with the upscaling of the sensor fabrication process [8]. GFETs can be found as top-gated, back-gated, or in a combined geometry, for the detection of a variety of molecules/biomolecules and ions [3], including pH [9], glucose [3], deoxyribonucleic acid (DNA) [3], proteins [5], and hormones [3].

Graphene is a zero bandgap material that generates electrons and holes under an applied transverse electric field, which is a process known as electrostatic gating [4]. Therefore, for positive or negative gate voltages, an electron or hole drain current is generated, with a minimum value at the charge neutrality point (CNP), where graphene is considered non-doped [4,10]. In a top gate GFET, a liquid electrolyte can replace the commonly used solid dielectrics (electrolyte-gate GFET, EG-GFET), which is usually a thin Al_2_O_3_ or SiO_2_ film [6]. In this configuration, the gate potential falls in the nanometer-thick electrical double-layers (EDL) forming at the solid–electrolyte interfaces, providing a high gate capacitance to operate the transistor at very low voltage. The possibility of detecting small changes in GFET transfer characteristics due to minute changes in the interfacial charge distribution that modulates the gate capacitance paves the way for ultrasensitive biosensing. Thus, EG-GFETs are ideal candidates for molecular sensing with electronic transduction [10,11].

**Figure 1:**
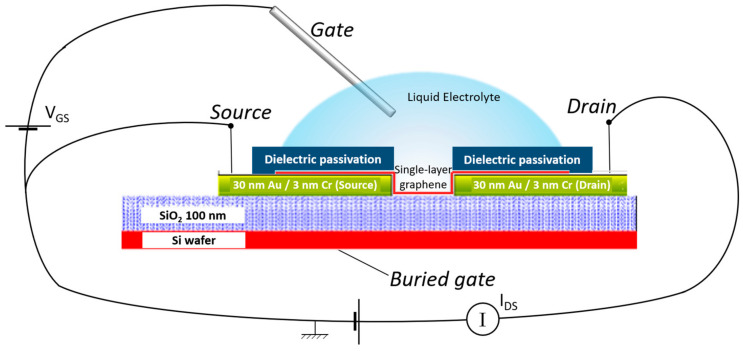
Working model of a typical graphene field-effect transistor in which an liquid electrolyte replaces the commonly used solid dielectrics (EG-GFET). A fixed voltage is applied between the source and drain contacts, and the current output is measured as a function of the gate-source voltage. A dielectric passivation is applied on top of the source and drain contacts to avoid interaction of the electrolyte at these regions to improve signal stability. V_GS_—gate-source voltage, I_DS_—drain-source current.

EG-GFETs are commonly built without the reference gate electrode (Figure 1), which is then added as an external, cumbersome metallic wire (made of gold, platinum, or silver) used for measurements [11]. Making the gate electrode part of the wafer, coplanar with the source, and drain electrodes allows for planar technology single-lithographic mask contact fabrication as well as design flexibly regarding the transistor layout [6,11].

When designing EG-GFETs, it is crucial to consider their application, e.g., the device’s environment, to decide design and fabrication steps. Electrolyte gating implies using liquids on top of semiconductor materials that typically degrade upon contact with aqueous solutions. Moreover, both semiconductor and dielectric materials are prone to host ions that diffuse inside their lattice, degrading the materials or rendering electronic measurements unstable. Therefore, the passivation of all surfaces in contact with the electrolyte, except graphene, is recommended to prevent/minimize such effects [11]. The passivation layer can be done by coating the wafer surface with photoresist [11], epoxy resin [12], or silicon nitride (SiN_x_) and silicon oxide (SiO_2_) layers [13,14]. An adequate protection strategy allows us to place the electrolyte over a large number of devices without cross-talking [15], reduce leakage current [12], reduce drift due to ion diffusion, and prevent damage of the metallic electrodes [15]. Thus, when designing a biosensors process, it is essential to consider permeability to salts and solvents to choose the passivation layer material. If polymers are chosen, they must be resistant to saline solutions [15] to avoid degradation upon exposure to biological solutions. If silicon-based materials are used, one must consider that SiO_2_ is much more permeable to ions than SiN_x_. A higher permeability may allow ions from the test solution to reach the current lines and cause drift, cross-talking between electrodes, or corrosion of the metallic contacts [15].

While using epoxy resins such as SU-8 negative photoresist provides a relatively inexpensive and straightforward process, it imposes a limit on the use of strong solvents in contact with the fabricated devices. For example, dimethylformamide (DMF) as a graphene cleaning agent after wafer fabrication and as a solvent in the graphene’s non-covalent functionalization [16] would not be allowed. Although DMF does not dissolve SU-8, it degrades and unseals continuous films [17]. We observed that DMF damages the direct-write epoxy resist mrDWL1x, promoting its release from the surface and re-deposition elsewhere, which renders the device unusable [18]. Passivation using SiO_2_ or SiN_x_ layers allows exposing the devices to all solvents without compromising the passivation. However, it implies more complex lithographic and etching processes, thus increasing the fabrication cost. In conclusion, it is of paramount importance to keep in mind the application of the EG-GFET when designing the passivation of current lines.

An important consideration is to decide at which point of the fabrication process the passivation layer should be added. Overall, lithographic processes involving aggressive patterning steps (e.g., dry etches, lift-off processes) should be avoided once graphene is transferred onto the wafer since they increase the amount of residues left on graphene [6]. Therefore, inorganic passivation layers should be patterned before graphene transfer. However, this leaves the source and drain contacts unprotected, since they receive graphene in a subsequent step. Leaving those contacts exposed to the electrolyte contributes to signal instability due to varying electrolyte potential during gate voltage sweeps [11] and device-to-device variability due to uneven coverage of the large electrode area with graphene, namely at its edges. In summary, the passivation layer’s optimized design recommends that it is added only after graphene transfer, protecting all surfaces except the graphene channel and the metal gate electrode, which need exposure to the electrolyte. However, this collides with the earlier stated principle of avoiding lithography of hard layers in the presence of graphene.

Passivation based on photoresists is done in a relatively straightforward process, using one lithographic step only followed by development in solution (a mild process) and sometimes a final bake to harden the layer. However, for passivation based on SiN_x_/SiO_2_, the process implies more aggressive steps, e.g., plasma-based etching or sputtering depositions that can affect graphene integrity [6]. Some authors avoid dry etching steps by patterning the passivation layer via wet etch using a strong basis or acid [19]. However, chemically dangerous solutions (e.g., buffered hydrofluoric acid (HF)) should be avoided for safety. Moreover, when performing wet etch, it is common to observe an undercut, promoting the passivation layer’s peeling. An alternative proposal, tested in this paper, is the combination of the two strategies, avoiding dry etch directly on graphene (partial dry etch) and reducing the times required for wet etch to limit the undercut effects.

Dependence on wet etch processes always limits reproducibility and process upscaling [13]. There are additional constraints that need to be met when considering dry etching on graphene. We suggest using a stopping layer on the graphene active regions preceding the passivation layer’s deposition in this work. The stopping layer serves two purposes: protect graphene during dry etching and provide a dry etch-resistant surface signaling the process’s end. For the deposition of the stopping layer, atomic layer deposition (ALD), thermal evaporation, and sputtering can be considered. ALD, owing to its very low deposition rate, can only be deposited in very thin layers. The ALD of aluminum oxide is useful for the thin gate dielectric in transistor structures. However, because graphene is hydrophobic, the ALD of aluminum on graphene must be preceded by an evaporated aluminum layer, which turns into an oxide layer by natural oxidation and presents a good surface for the ALD growth [20,21]. However, then, for a thin aluminum oxide stopping layer, there is no necessity to use ALD provided physical vapor deposition systems are used under conditions that minimize graphene damage. Here, we chose the stopping layer material from those possible to grow under milder sputtering conditions.

Another critical step in EG-GFET fabrication is the graphene transfer. For applications requiring electronic grade material, there are few choices available beyond mechanically exfoliated graphene from highly oriented pyrolytic graphite (HOPG), Chemical Vapor Deposition (CVD) being one of them [22]. Still, when a large area is required (wafer-scale fabrication), mechanical exfoliation is not an alternative. Graphene grown by thermal CVD on copper catalysts or the Si face of SiC wafers are currently the preferred solutions to provide high-quality graphene over a large area [23]. The first approach is by far the most popular because of its low cost and high versatility [23], allowing the transfer of graphene onto virtually any substrate type. If precise control of the oxygen supply to the Cu surface during growth is put in place, huge crystal size and carrier mobility are achieved [24]. Graphene grown on SiC wafers has the advantage of growing natively on a transparent insulating substrate and enables transfer-free fabrication of optoelectronic devices [25]. However, monocrystalline SiC substrates are too expensive to be used in large-scale production, and graphene grain size is limited by the atomic terraces’ sub-micrometer width, unlike CVD graphene.

Although wet transfer methods of CVD-grown graphene give the most promising results for the large-area fabrication of GFETs, there are still challenges related to surface contaminants [23,26] often overlooked in the biosensing field [27]. After graphene transfer and patterning, visible residues of different origins (polymethylmetacrylate (PMMA), metal residues) are almost always found on the device surface. Residues on the sensing surface hinder functionalization and promote the non-specific binding of biomolecules in undesired areas [27]. It is crucial to search for new strategies to improve surface cleaning after graphene transfer and patterning. After etching graphene from undesired surfaces (e.g., from the gate electrode), the residues attached to it (mainly metal contaminants) are not entirely removed from the surface, bringing reproducibility usability issues to the biosensing device. In this work, we address strategies to improve surface cleaning after graphene patterning. Those issues are critical in the fabrication of EG-GFET biosensors with the highest sensitivity and lowest limit of detection.

## 2. Materials and Methods

### 2.1. Materials

Materials used for microfabrication of EG-GFETs are listed in Table 1.

### 2.2. Characterization Techniques

#### 2.2.1. Raman Spectroscopy

Confocal Raman spectroscopy (WITec Alpha 300R, Ulm, Germany) is used to confirm single-layer graphene presence in transferred samples and detect variations in graphene quality after microfabrication processes. Raman data analysis is done using WITec Project FOUR+ software, Ulm, Germany. All samples are analyzed using a 532 nm Nd:YAG laser for excitation. The laser beam with power in the range 2 to 3 mW is focused on the sample by a ×50 lens (Carl Zeiss, Jena, Germany), and the spectra are collected with a 600 groove/mm grating using 5 acquisitions each with 2 s acquisition time. Within each experiment, the laser power is fixed to allow comparison of the acquired data.

#### 2.2.2. Scanning Electron Microscopy/Energy-Dispersive X-ray Spectroscopy

Scanning Electron Microscopy (SEM), FEI NovaNanoSEM 650, Hillsboro, OR, USA, is used to study surface features and energy-dispersive X-ray spectroscopy (EDX) analysis. SEM images are collected in the secondary electron imaging mode at a 5 mm working distance and an operating voltage of 5.0 or 10.0 kV, depending on the materials to be analyzed and the probing depth desired. EDX acquisition using Oxford Instruments INCA software, Oxfordshire, UK, is performed on selected regions from SEM images to confirm surface chemical composition and detect surface contaminants after completing the fabrication process.

#### 2.2.3. Mechanical Profilometer

KLA—Tencor P-16 Surface Profiler, Scotia, NY, USA, is used to measure photoresist films’ step height after O_2_ plasma-based etching steps.

#### 2.2.4. Optical Interferometer

OPM Nanocalc—Optical Profilometer/Interferometer, Ettlingen, Germany, is used to estimate SiO_2_ and SiNx films’ thickness, using a deuterium–tungsten halogen light source. The thickness is determined by fitting to a model using reflected light between 40 and 800 nm.

#### 2.2.5. Optical Microscope

Nikon Ni-E optical microscopes, Tokio, Japan, are used to visually assess the sample surface after each of the fabrication steps.

#### 2.2.6. Graphene EGFET Electrical Characterization

For acquisition on the transfer curves of the fabricated EG-GFET samples, the source and drain contacts of each transistor are connected to a Keithley 4687 Picoammeter, Cleveland, OH, USA, which applied a constant (1 mV) source–drain voltage (V_SD_), and the source–drain current (I_SD_) is measured. The high-impedance gate circuit is formed between gate and source contacts. It is biased by applying a voltage (V_GS_) by a Keithley 2400 source meter, Cleveland, OH, USA, which is programmed to apply voltage ramps from 0.2 to 1.2 V in steps of 0.01 V. All the measurements were performed using 10 mM phosphate buffer as gate electrolyte to close the circuit.

For full-wafer characterization, an automated wafer probe station is used. The graphene channels’ resistance is analyzed by applying a constant voltage between source and drain (1 mV) and measuring the channel’s current, using a Keithley 2400 source meter. No liquid electrolyte is added to the devices, and no gate voltage is applied (the gate contact is left floating). Channel resistance values by the device are obtained by processing the current measurements using MATLAB scripts.

## 3. Results and Discussion

### 3.1. How to Transfer and Pattern Graphene Leaving a Clean Wafer Surface

In this study, graphene is grown by CVD in a hot-wall reactor (First Nano ET3000, New York, NY, USA) on high-purity Cu catalysts and transferred (see Supplementary Material, Section S1.2.3). The process is optimized to achieve monolayer films with low defect density, as shown by the Raman spectra in Figure S1. Graphene is transferred to the final substrate using a temporary polymeric substrate (PMMA) as described in Section S1.2.3 of the Supplementary Material.

Graphene transfer and patterning are crucial steps in the fabrication of graphene devices. Except for automated, continuous transfer systems [28], which are not universally available yet, the wet graphene transfer limitations are mostly user-related, requiring a trained hand to achieve reproducible results. At least two kinds of transfer-borne residues can come from the transfer process. Apart from polymeric residues, there can be metal residues made of copper from the foil or iron precipitates from the FeCl_3_ solution that stay attached to the graphene film’s bottom side that stick to the surfaces to which graphene is transferred, as shown in Figure S2. Precipitates can be reduced by using a metal-free etchant such as ammonium persulfate or dissolving the iron using the HCl solution. Air bubbles present in the copper foil can lead to undissolved copper clusters after the dissolution process. Using fresh FeCl_3_ solutions and sequential dissolutions can improve the removal of Cu clusters. Regardless, small Cu atomic clusters and ions are always observed in the surfaces after graphene transfer, as shown by PIXE studies [29]. Those particles may not influence graphene’s surface processes, since they are performed on the top side (the residues are mostly trapped at the interface between the substrate and graphene) but are transferred to other exposed wafer surfaces. In our design, where the top gate electrode is coplanar with the transistor channel, the exposure of this electrode during transfer leads to adsorption of the residues on the gold surface, as evidenced in Figure S2d. This contamination is also a source of parasitic signal because exposure to biological solutions leads to non-specific protein adsorption at the particle sites. In particular, if such sites are located on the gate electrode, changes in the voltage drop at the gate–electrolyte interface are observed [30,31]. This effect changes the biosensor’s concentration-dependent behavior to a random behavior hindering interpretation, optimization, and use of the device.

#### 3.1.1. Dry Etching

**Figure 2:**
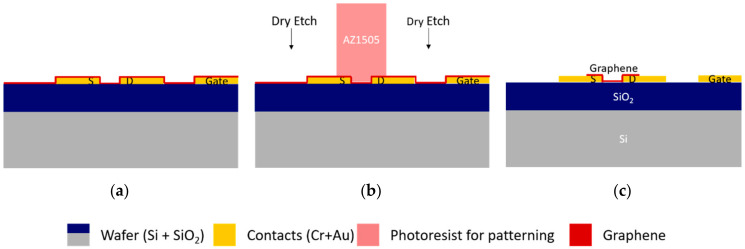
Graphene patterning, after wet transfer, by standard O_2_ plasma etching. (**a**) After wet transfer, graphene covers all the surfaces of the devices. (**b**) Use of AZ1505 photoresist to protect the channel, source, and drain during O_2_ plasma etch of excess graphene. (**c**) Patterned sample with graphene covering the source and drain contacts and the channel between them.

The most common way to pattern graphene is reactive ion etching, using an oxygen plasma that readily attacks and removes carbon (Figure 2). For dense patterns or smaller features where mask transfer accuracy is critical, a plasma technique with some ion bombardment level is necessary, such as commonly used inductively-coupled plasma sources (ICP). However, electron cyclotron resonance remote plasma sources (ECR) are suitable for isolated and micrometer-sized features. When using dry etching methods, the patterning material, photoresist, must be considered carefully to ensure it sustains the etching process while being easily removed after patterning, leaving few residues on graphene. The etch process’s critical parameters are the radio-frequency (RF) power and the O_2_ partial pressure since they influence the etching efficiency and the exposure time that the photoresist can sustain. However, the process is dependent on the size of the etched area. For example, etching small substrates with a few tens of square centimeters is very different in etching time and uniformity than etching a 200 mm-wafer due to changes in the plasma current at the surface of the sample [32,33].

**Figure 3:**
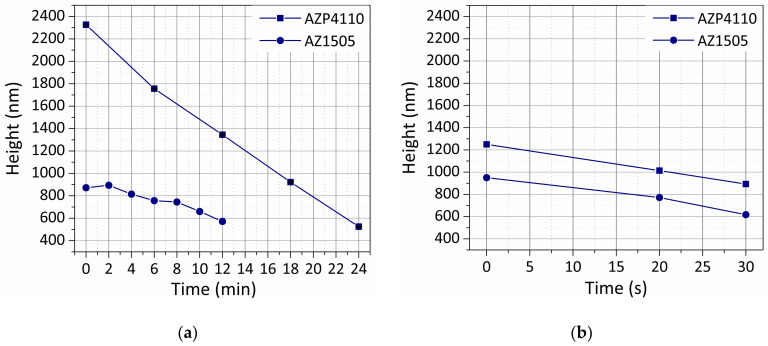
Thickness variation of different positive photoresists as a function of exposure time to O2 plasma using electron cyclotron resonance (ECR) (low power) plasma source (**a**) and inductively-coupled (ICP) (high power) plasma source (**b**).

Two positive photoresists (AZ1505 and AZP4110, see Table 1) were exposed to plasma etching in a barrel reactor ECR plasma asher and an ICP reactive ion etching system. We first obtained conditions suitable for graphene etching in the two systems; then, we obtained the resist etch rate. To this goal, we measured the remaining thickness of 25 μm × 75 μm isolated resist features using a mechanical profilometer (KLA—Tencor P-16 Surface Profiler) in between etching steps as a function of the total etching time (Figure S3 (profiles) and Figure 3). The figures for photoresist height variation with O_2_ plasma time indicate that the photoresist is etched much faster when using the ICP O_2_ reactive ion etching (RIE) (Figure 3b) than when using the ECR O_2_ plasma source (Figure 3a). ECR shows different etch rates between the two photoresist masks. The high-power (1200 W) ICP leads to a ≈700 nm/min etching rate for both photoresists. The low-power (230 W) ECR asher, with a sample temperature lower than 100 °C, leads to an etch rate of ≈30 nm/min for AZ1505 and of ≈70 nm/min for AZP4110.

Considering the etching times allowed by both methods, samples with pre-patterned contacts and graphene are patterned. For the ECR plasma source, the sample is coated with AZP4110 at 2200 nm (nominal thickness) to maximize the etch time. The sample is etched for a total time of 18 min divided into 6 min etching periods (to allow better temperature control of the sample). For the ICP plasma source, the sample is coated with AZ1505 at 1035 nm (nominal thickness) and etched for 10 s. According to the values in Figure 3, both samples should end up with approximately the same photoresist thickness, ensuring proper protection of the graphene channels. The optical photographs in Figure S4 show efficient graphene removal for both methods. However, transfer-borne residues are also visible with high density.

High power processes (above 1000 W [34]) are highly effective in removing graphene, with 10 s being enough for full 200 mm-wafer patterning. However, removal of the photoresist mask is also swift, on the one hand, and high temperatures can be quickly achieved, increasing the risk of photoresist burning, on the other. Consequently, the photoresist thickness and time of exposure to high power plasma needs optimization to ensure that the photoresist endures the process without being burnt/removed. The ICP O_2_ plasma is also expected to remove graphene transfer-related residues (e.g., PMMA) from the surface. However, the optimal time for graphene patterning without removing the photoresist is usually not enough to achieve good surface cleaning. The use of low ECR-power O_2_ plasma requires increased time of process. Still, it allows for improved control of the system temperature, improving photoresist stability, reducing at least 10 times in the photoresist etch rate. With longer times, organic residues’ removal from the surface is also improved, and graphene is efficiently etched from a full 200 mm wafer in less than 18 min. Therefore, in the following, ECR-O_2_ plasma is preferred whenever the dry etching of graphene is required.

The limitation for ECR-O_2_ plasma etching is removing metallic particles or other non-organic transfer-borne residues. These residues that come adsorbed to the backside of graphene upon transfer are not removed by O_2_ plasma and hinder device performance, as detailed above. A more elaborate strategy is necessary, preventing direct contact between residues and chip surface. In the next section, a lift-off process is studied to limit the wafer surface’s contamination by debris resulting from the graphene transfer process.

#### 3.1.2. Lift-Off Based Transfer

**Figure 4:**
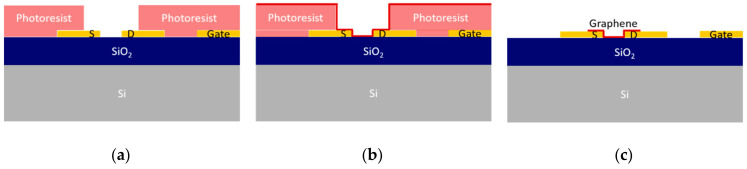
Lift-off assisted graphene transfer and patterning. First, the sample is patterned by optical lithography to leave the source and drain electrodes and channel exposed (**a**). During graphene transfer, the remaining surface is protected with photoresist (**b**) that is removed along with PMMA, leaving the surface of the devices clean (**c**). Graphene is represented as a red line.

A lift-off inspired process could be an alternative to the standard dry etch patterning, allowing minimum contact of graphene with unnecessary/undesired regions of the surface and achieving a cleaner fabrication process. It is our experience that when transferring graphene over a surface with relatively steep steps (above 100 nm), the film likely breaks and tears apart (crack propagation) at the step region. Therefore, using a lift-off technique to transfer graphene only to the wafer regions where transistor channels form should be possible. For lift-off assisted wet transfer, a sample with gold contacts is spin-coated with photoresist (AZ1505 1035 nm or AZP4110 2200 nm) and patterned by optical lithography. The transfer region is left unprotected, as schematically shown in Figure 4.

Graphene transfer is performed as described in the Supplementary Materials (Section S1.2.3) using PMMA as a transfer substrate. The samples are annealed in a low vacuum (desiccator) for 2 h after the transfer to improve graphene adhesion. Photoresist dissolution coincides with PMMA removal. After PMMA removal, the samples are observed by optical microscopy to assess the lift-off process’s success.

The optical images in Figure S5 clearly show that the lift-off process quality is dependent on the size and distribution of the features patterned on the surface. Although a systematic breaking of the graphene film in the outer edges of features with sub-millimeter area (≈0.4 mm2) in size is observed (Figure S5b), in smaller features (≈0.012 mm2), the breaking occurs randomly in regions out of the delimited pattern (Figure S5a, red rectangles), creating floating parts of the film that end up on top of the region of interest (Figure S5a). For graphene features separated by short gaps (less than ≈20 μm), the graphene film does not break between the gaps even with 2.2 μm height steps, keeping its structural integrity instead of adhering to the surface after the photoresist removal.

**Figure 5:**
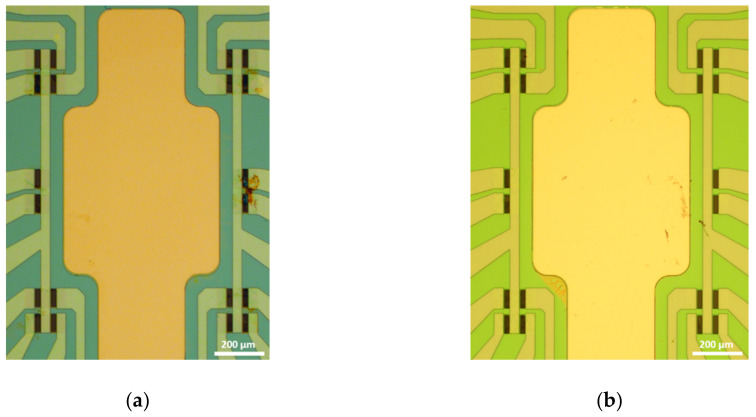
Optical image of the final surface achieved after graphene lift-off-based transfer and patterning (**a**) and patterning by dry etch (**b**). The scale bars represent 200 μm.

The clear advantage achieved with the lift-off strategy is the final quality of the device surface. Figure 5a shows a cleaner gold surface when compared to the final surface of chips fabricated by dry etch techniques (Figure 5b), but it is only suitable for designs where the devices are separated from each other by at least some millimeters.

#### 3.1.3. Combined Approach

**Figure 6:**
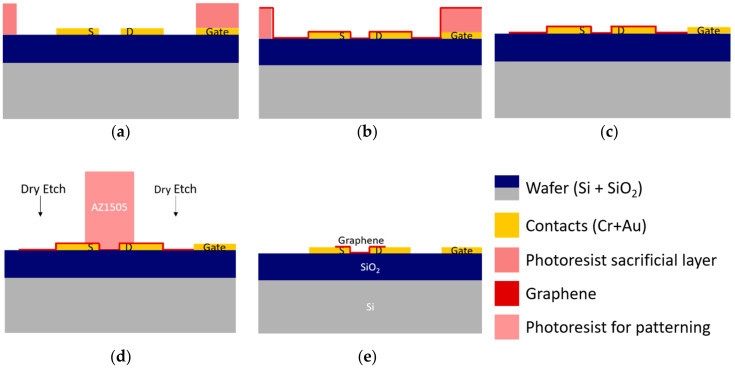
Graphene transfer and patterning using a combined lift-off and O_2_ patterning method. (**a**) A photoresist sacrificial layer is used to promote initial patterning of graphene and prevent the accumulation of transfer-related residues at the gate electrode. (**b**) During the transfer of graphene, the 2D material breaks at the borders of the photoresist. (**c**) Sample after initial patterning. (**d**) Standard O_2_ plasma etch for fine-tuning of graphene patterning. (**e**) Patterned sample with graphene covering the source and drain contacts and the channel between them.

Based on the results of Section 3.1.1 and Section 3.1.2, particularly concerning the downsides of the dry etch (surface dirtiness) and the lift-off method (lack of patterning precision), a new combined strategy to improve the process is envisaged as shown in Figure 6. As breaking occurred at the expected region for extensive features, this can be used to perform a pre-patterning of graphene that keeps the relatively large (≈0.7 mm2) in-plane gate electrode clean after the transfer. Then, dry etching is used for the fine patterning of graphene in the channel area, leaving the pristine gate electrode free of residues. An extra lithographic step is added, as shown in Figure S6a, consisting of a large square area around the region of interest where graphene can contact and adhere to the surface. Inside that square, the photoresist protects the gate electrode, keeping it as clean as possible. After photoresist and PMMA dissolution, the graphene channels are then patterned by RIE.

A test sample was prepared using 2200 nm AZ4110 photoresist for the lift-off assisted graphene transfer. After transfer and PMMA removal, inspection by optical microscopy accesses the quality of the patterning. Although the features were large and well separated, Figure S6b shows that crack propagation in graphene occurred only systematically in the photoresist mesa’s outer limits, i.e., where distances between the photoresist steps were in the order of millimeters. In the region of interest, i.e., the gate electrode, the photoresist protection was dissolved without breaking the graphene film. Therefore, the combined strategy was abandoned, since graphene’s mechanical properties make lift-off a technique hard to master for patterning this material, leading to the accumulation of residues originated from the gate electrode’s wet transfer process surface, compromising the performance of the device for biosensing.

#### 3.1.4. Pre-Transfer Sacrificial Layer

**Figure 7:**
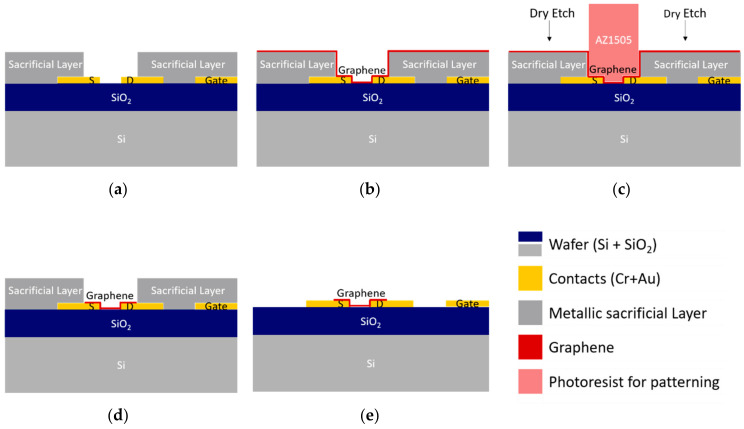
Pre-transfer sacrificial layer to protect the chip surface from residues. (**a**) Preparation of the sacrificial layer via lift-off, leaning only the channel region and source and drain electrodes exposed for the graphene transfer. (**b**) Graphene transfers over the protected devices, where it only contacts the actual surface where desired. (**c**) Graphene patterning using O2 plasma (dry etching). (**d**) The exposed sacrificial layer is removed by wet etch, removing the residues left on the transfer process’s surface. (**e**) Finalized graphene transfer and patterning process, leaving the gold surfaces free of residues.

The previous sections show that lift-off patterning graphene films produce inferior results compared to patterning standard materials by the same technique. Consequently, a novel methodology is developed, as schematically shown in Figure 7. A hard mask is used, not to pattern graphene but to protect the substrate during graphene transfer and patterning by O_2_ plasma using a photoresist mask. Any contaminants not removed by the oxygen plasma are removed together with the hard sacrificial mask. The contaminants possibly deposited with graphene on the source and drain are not avoided. Still, they do not significantly affect the device performance, since they are not in contact with the biological solutions.

The sacrificial layer should withstand the standard transfer process and patterning of graphene, and it should also be easily removed (e.g., dissolution), leaving the graphene intact. With that in mind, it was designed as follows: first, a buffer Al_2_O_3_ film was sputtered for physical separation of the Au surfaces from the sacrificial metal layers, avoiding inter-layer diffusion. Films with 10 and later 20 nm of Al_2_O_3_ were used to ensure this layer’s stability during aluminum etch (the etchant is the same for both Au and Al_2_O_3_). The next layer in the stack is AlSiCu, the protective layer that avoids residues to deposit on the devices’ surface. The capping layer is TiW(N) to allow all lithographic processes (the photoresist developer quickly dissolves AlSiCu and is too reflective for precise laser exposure). Al_2_O_3_ may replace TiW(N) as a capping layer to avoid repeated use of H_2_O_2_ to etch TiW(N), which increases the risk of damaging graphene (see Section 3.2.2).

At the point of the fabrication sequence where the sacrificial layer is added, only the contacts have been patterned, and so a standard lift-off can be used with no risk of damaging underlying layers. Thus, after a lithographic step that leaves the photoresist protecting the source, drain, and channel, the sacrificial layer stack is deposited by sputtering followed by lift-off using acetone and ultrasonic bath. Next, graphene is transferred by wet transfer (Figure 7a), and PMMA is removed with acetone (Figure 7b). Then, graphene is patterned using three cycles of 6 min of a low power ECR-O2 plasma (Figure 7c and Section 3.1.1). Wet etching of the sacrificial layer follows, using the photoresist from the previous step to protect graphene. H_2_O_2_ removes TiW(N), or AZ400k 1:4 developer removes Al_2_O_3_, depending on which material was used as the capping layer. AZ400k can etch both Al_2_O_3_ and AlSiCu, so it is used to etch the adjacent AlSiCu layer. The etch rate of AlSiCu is 10 times higher than that of Al_2_O_3_, so the etch time is adjusted to preserve the Al_2_O_3_ buffer layer. However, optical images after the process (Figure S7d, red circled regions) show the total removal of the Al_2_O_3_ film in certain areas due to the over-etch required for removal of the AlSiCu film (non-homogeneous etch). This forces stopping the fabrication process, since gold is no longer protected.

A further increase in Al_2_O_3_ thickness could improve this result. However, it is difficult to envision because Al_2_O_3_ needs to be later selectively removed (before the dielectric passivation deposition, Section 3.2) by wet etching using an alkaline solution, causing unwanted dark erosion of the AZP4110 photoresist mask. Consequently, 20 nm seemed to be the maximum Al_2_O_3_ thickness compatible with this process. As an alternative, a thin layer of TiW(N) was deposited between the buffer layer of Al_2_O_3_ and AlSiCu. In this way, the wet etch of AlSiCu is accomplished with no attack on the Al_2_O_3_.

**Figure 8:**
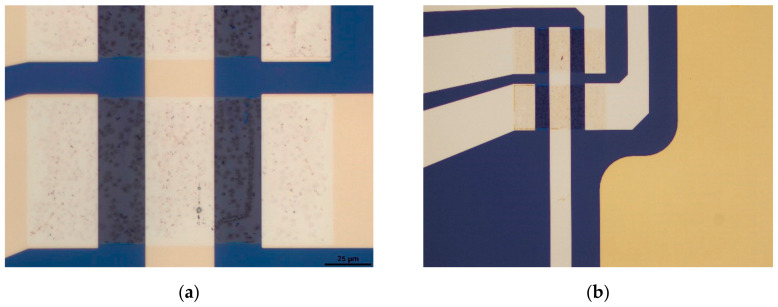
Optical microscope images reveal well-defined patterning of graphene (**a**) and clean surfaces after removing the sacrificial layer (**b**).

Following the above process, a new sample is prepared and observed with the optical microscope revealing spotless surfaces with well-patterned graphene transistors, as seen in Figure 8, which shows that the process is successful.

A final comment on the use of TiW(N) or Al_2_O_3_ as a capping layer: partial removal of the sacrificial layer occurs upon graphene transfer (Figure S8) due to HCl residues that are trapped between graphene and the Al_2_O_3_ layer. If the graphene is transferred in a single step, this is irrelevant, and the Al_2_O_3_ capping layer can be used. However, if the wafer coverage is attained by multiple transfer of graphene patches, then TiW(N) should be chosen, because the HCl residues do not degrade it. Then, it should be as thin as possible to reduce the photoresist/graphene exposure time to H_2_O_2_ during TiW(N) etch.

For these reasons, the sacrificial layer stack was defined as TiW(N) 5 nm/AlSiCu 100 nm/TiW(N) 15 nm. The wet etch process to remove it was H_2_O_2_ 30% 150 s, then AZ400K 1:4 240 s, H_2_O_2_ 30% 50 s. This stack proved useful in avoiding residues in the gold gate electrode since, after this process, the Al_2_O_3_ film on top of gold is particle-free.

### 3.2. Fabrication of the Dielectric Passivation Layer

Before designing the passivation layer, several points need consideration. First, consider the final use of the devices. In our case, a clean biosensing interface is obtained by the use of strong solvents (e.g., DMF) [16] that attack polymeric materials, including photoresists. For example, we observed that DMF damages the direct-write epoxy resist mrDWL1x, promoting its release from the surface and re-deposition elsewhere, which renders the device unusable [19]. Second, along all stages of bio-functionalization, bio-recognition, and transducing, the devices are exposed to salt-containing solutions (e.g., phosphate buffer, PB), which recommends that the top layer of the passivation has minimum permeability to ions. With these considerations in mind, a protective coating consisting of a stack of five alternated SiO_2_ and SiN_x_ films with a total thickness of 250 nm is designed, starting with a layer of SiO_2_ to take advantage of the good adhesion of SiO_2_ to graphene and the substrate, and terminating with SiNx to take advantage of the superior impermeability of SiN_x_. The multilayer, deposited by plasma-enhanced CVD (PECVD), hinders native nucleation centers and breaks the propagation of defects through the layers, creating tortuous diffusion paths for water and other small molecules [35,36]. All tests are performed after this rugged, chemically stable passivation layer is in place.

**Figure 9:**
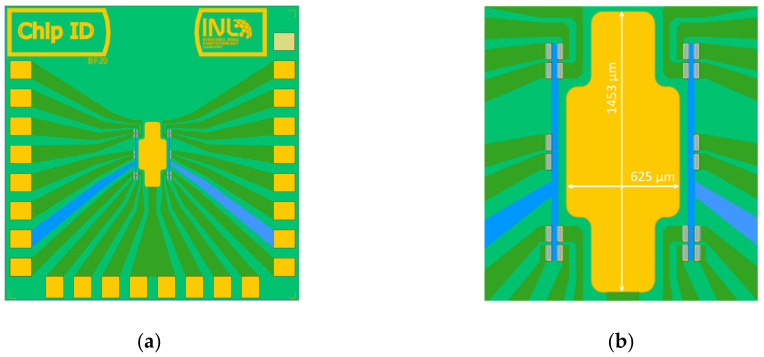
Layout for graphene-based biosensors. Layout total size is 6.75 mm by 6.75 mm (**a**), composed of 20 transistor channels with individual drain electrodes, a common source electrode for each 10 transistors, and a common in-plane gate electrode (**b**).

For testing, samples of 4 × 4 cm^2^, each containing 36 chips of 20 EG-GFETs, were fabricated as described in Section S1.2.2 of the Supplementary Material. Figure 9 shows the general design of each chip (Figure 9a) and the disposition of the graphene channels (gray) and gate electrode (yellow) (Figure 9b).

#### 3.2.1. Combined Reactive Ion Etching and Wet Etch Strategy

Some reports refer to dry etching methods [37] to open access vias to graphene in the passivation layer, while others prefer wet etch methods [19] based on HF. Although dry etch is a highly controlled process and very efficient in the etching of dielectric materials, its use directly in a layer adjacent to single-layer graphene would damage graphene due to the plasma-based process. Although less controlled (it often produces an undercut under the edge of the patterned zone), wet etch can be used without damaging graphene. Therefore, to open the access vias on the passivation layer on top of graphene without compromising the graphene quality, a mixed strategy combining dry and wet etching techniques was proposed and tested.

**Figure 10:**
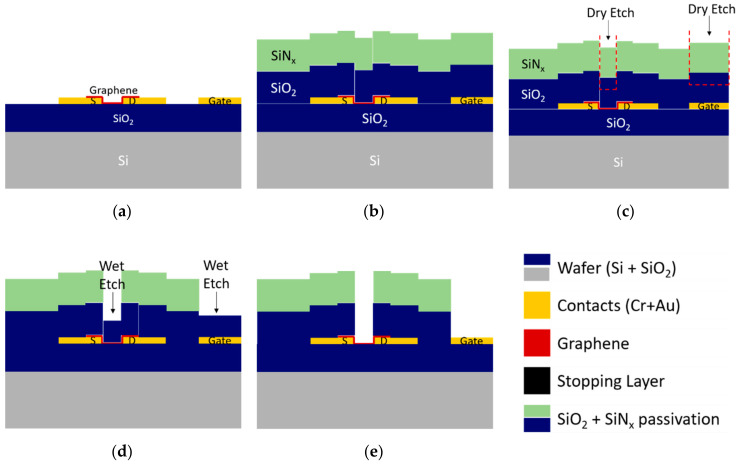
Patterning of dielectric passivation by combined reactive ion etching (RIE) and wet etch methods. (**a**) Sample after graphene transfer and patterning, ready for dielectric passivation. (**b**) Deposition by PECVD of SiO_2_ and SiN_x_ dielectric passivation. (**c**) Dry etch patterning of the passivation, reaching the SiO_2_ layer. The remaining thickness allows graphene to be protected during RIE and will be removed by wet etch. (**d**) Wet etch with KOH at 60 °C to remove remaining SiO_2_ from graphene and the gate electrode. (**e**) Final device with only the graphene channel and the gate electrode exposed for contact with the electrolyte.

First, dry etching is used to open the top layer of the passivation stack (Figure 10c partially), and then wet etch with a strong basis or acid opens the remaining layers of the passivation stack (Figure 10d). Many acidic wet etching solutions, such as piranha solution or aqua regia, are oxidizing agents and react strongly with organic Si compounds. Thus, they must be avoided. Phosphoric acid (often used as Al etchant) is known to create pores in graphene [38], and solutions based on hydrofluoric acid (HF), which is also a graphene dopant, should be avoided for safety. On the other hand, base solutions, such as KOH and tetramethylammonium hydroxide (TMAH), are not known to attack graphene and are therefore good candidates for the wet etch of the dielectrics. Thus, KOH 1 M was used as the etchant for the second step of the passivation patterning.

The details of this study can be found in Supplementary Material and Figure S9. The results indicate that the wet etch step is systematically causing the SiO_2_/SiNx stack delamination, as shown in Figure S10. It is hypothesized that delamination could be caused by the undercut of the SiO_2_ film under the SiN_x_ during the KOH wet etch process, promoting the top layer’s stress release by peeling (see Figure S10) and rendering the devices unusable. An approach to overcome this issue could be using HF vapor in a vacuum instead of liquid etchant. Still, when up-scaling to the wafer level, the process steps’ economy and homogeneity over a large area are essential. HF vapor etching is typically used for the complete removal of oxides with large over-etch times. In contrast, the use for patterned etching is highly non-homogeneous due to the anisotropy of the vapor flow, which is an effect that is difficult to compensate when removing relatively thin films (<100 nm) [39]. Therefore, a satisfactory solution, combining wet and dry etching, could not be found for the graphene EG-FET fabrication.

#### 3.2.2. Stopping Layer-Assisted Reactive Ion Etching of Dielectric Passivation

As the combination of dry and wet etch presents significant limitations due to the wet etch step, other alternatives were sought in the literature to achieve patterning of passivation layers through reactive ion etching (RIE) [37]. However, none of them discuss how the graphene surface can be protected during this process. For the deposition of the stopping layer, atomic layer deposition (ALD), thermal evaporation, and physical vapor deposition (PVD) can be considered. ALD, owing to its very low deposition rate, can only be deposited in very thin layers. ALD of aluminum oxide is useful as a thin gate dielectric for transistor structures, so this technique has been studied for GFETs. However, because graphene is hydrophobic, the ALD of aluminum on graphene must be preceded by a thin evaporated aluminum layer, which turns into an oxide layer by natural oxidation and presents a good surface for the ALD growth [20,21]. However, there is no real advantage in using ALD if another technique precedes it for a stopping layer composed of aluminum oxide. Therefore, physical vapor deposition systems are tuned to provide mild deposition conditions of relevant materials to minimize graphene damage.

**Figure 11:**
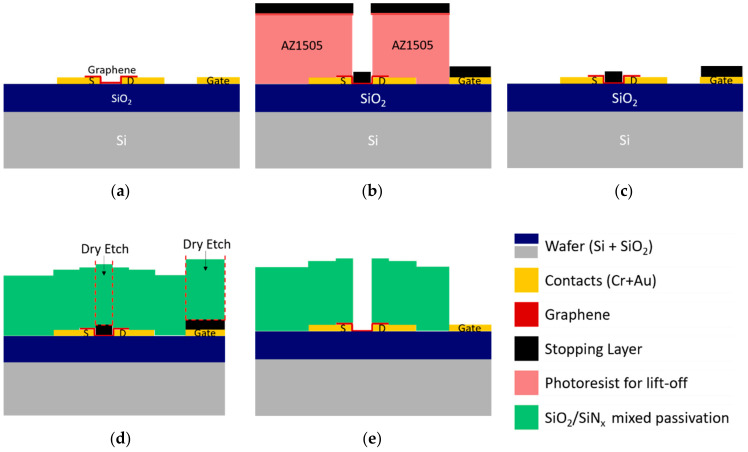
Patterning of the dielectric passivation by RIE using a sacrificial/stopping layer. (**a**) Sample after graphene transfer and patterning, ready for dielectric passivation. (**b**) Preparation of the metallic stopping layer by ultrasonication-free lift-off. (**c**) Stopping layer after patterning covering the graphene channel and the gate electrode. (**d**) Deposition by PECVD of SiO_2_ and SiN_x_ dielectric passivation and standard RIE patterning. (**e**) After wet, final device etch of the stopping layer, with only the graphene channel and the gate electrode exposed for contact with the electrolyte.

We propose a specific solution, consisting of the insertion of a stopping layer between graphene and the dielectric to be etched (see Figure 11). When a plasma etching is performed on graphene, a stopping layer must be prepared on top of it to prevent the etching from damaging graphene. For reactive ion etching of silicon oxide, the typical stopping layer is based on aluminum, either metallic or oxidized, which is not etched by fluorine chemistry. Still, graphene integrity must also be taken into consideration while building this layer. We performed the graphene integrity study as follows: six different materials (Al_2_O_3_, AlSiCu, TiW(N), Al, Cu, and Ni), ranging from metals to insulators, are sputtered (RF or DC with low and high power) on top of several graphene samples. After sputtering, a wet etch of those materials is performed at room temperature and with mild agitation for all samples. Different etching agents are used to match the material to etch: AZ 400k 1:4 for Al_2_O_3_, AlSiCu and Al, H_2_O_2_ 30% for TiW(N), and FeCl_3_ 0.5 M for Cu and Ni. The etching process is stopped when the samples seem clean. The graphene quality of these samples is analyzed by Raman Spectroscopy before and after sputtering and wet etch. The comparison of Raman spectra for all combinations shown in Figure S11 indicates that sputtering of most of these materials is very damaging to graphene. However, when sputtering Cu and Ni, the damage is not significant.

DC sputtering of AlSiCu and TiW(N) at 2 kW and 1 kW, respectively, and RF sputtering of aluminum oxide at 2.5 kW were found to damage or altered the material extensively. The deposition of oxide films, such as Al_2_O_3_, seems to induce the oxidation of graphene. The deposition of AlSiCu also induced many defects that could be originated by the high power plasma of the sputtering system with an additional contribution from mechanical damage during the dissolution of aluminum, where bubbling was observed. A similar situation occurs with TiW(N), where the etchant H_2_O_2_ 30% leads to the complete oxidation of graphene. Then, a low power (100 W) sputtering system with a sizeable target-sample distance of 30 cm (larger than in the system previously used) was tested. While the RF sputtering of pure aluminum lead to the damage of graphene after its removal, the DC sputtering of Cu and Ni did not induce a significant increase in the Raman defects peak (D mode: ≈1350 cm^−1^, Figure S11e,f).

Interestingly, these metals are also well-known catalysts for graphene growth, and their deposition on graphene was reported as healing graphene [40]. Once graphene is protected by a thin layer of Cu or Ni, further depositions of varied materials and higher powers can be successfully performed without damaging graphene. Thus, an effective stopping layer for dry etching of the passivation layer without compromising graphene integrity is achieved.

The next challenge for the preparation of the stopping layer is patterning. Patterning by wet etch should be avoided if we are to precisely control the features’ size, and patterning by standard lift-off is limited, since ultra-sonication is forbidden due to the graphene underlayer. Therefore, the lift-off process was changed as follows. Soaking the photoresist with a TMAH-based developer (AR 300.47, diluted 4:3 in water) before exposure creates a top hardened layer that behaves differently upon exposure and development. Development occurs faster in the remaining photoresist than in the hardened layer, producing “mushroom hat”-like features, or a negative profile, which creates a shadow during metal deposition and, therefore, facilitates lift-off. Since this feature forms at the top photoresist surface, immersion in a solvent peels-off this layer, where the sputtered materials are adhered. Testing this methodology on samples with pre-patterned contacts and graphene was highly effective and reproducible even at a wafer scale.

Based on the presented results, samples with pre-patterned gold contacts were prepared to test the passivation patterning process by dry etching using the best protective stopping layers. Samples with gold contacts and graphene are prepared as follows: the samples are coated with 600 nm of AZ1505 photoresist and soaked in the developer (AZ400k 1:4) previously to optical lithography. After lithography and development, each sample is sputter-coated with copper (Cu, 10 nm) or nickel (Ni, 10 nm), which is followed by aluminum alloy (AlSiCu, 30 nm) and titanium-tungsten (TiW(N), 5 nm). Then, the samples are immersed in acetone for 1 h at room temperature and then rinsed with an acetone wash bottle to promote the loose material’s release. The soaking method avoids sonication, preventing the damage/delamination of graphene. Next, the passivation layer is deposited as a stack of two 50 nm films of SiO_2_ and three 50 nm films of SiN_x_ with 250 nm of final thickness. The passivation stack is patterned using 1035 nm of AZ1505 photoresist and optical lithography, followed by RIE using previously optimized parameters. After removing the remaining photoresist, the channels and the gate electrode are released by wet etch of the stopping layer (AZ 400K 1:4 for AlSiCu and FeCl_3_ 0.5 M for Cu or Ni). The removal of the stopping layer was followed by EDX, allowing the final devices to be process contaminants free. The EDX spectrum in Figure S12a shows the stopping layer’s condition after patterning the passivation.

The RIE etching step etches away the SiO_2_/SiN_x_ passivation stack and the capping layer of TiW(N) and stops at the AlSiCu layer. The removal of AlSiCu by an alkaline solution (AZ400k) is confirmed in the spectrum of Figure S12b, where the peak of Al is significantly reduced. A small signature of Al is still visible due to the protective Al_2_O_3_ that is beneath the Cu of the stopping layer. The spectrum confirms the removal of the Cu by FeCl3 0.5 M in Figure S12c by the absence of any Cu related peak. The surface’s final status can be observed in Figure S12d, after removing the protective Al_2_O_3_ from the gold surfaces and showing no evidence of significant contaminants at the surface of the device.

**Figure 12:**
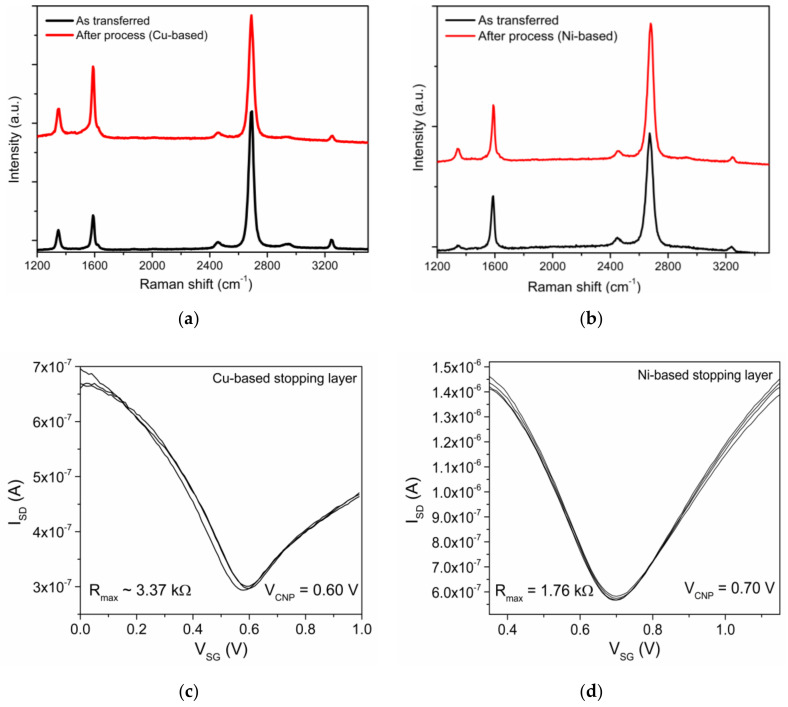
Characterization of EG-GFETs fabricated with dielectric passivation by Raman spectroscopy before sputtering of the stopping layer (black) and after complete processing (red) using Cu (**a**) or Ni (**b**) as the basis. The electrical characterization of the respective EG-GFETs in deionized (DI) water shows transistor behavior of the devices and similarity in the transfer curve characteristics both for a Cu-based process (**c**) and Ni-based process (**d**).

Confirming the results shown in Figure S11, the Raman spectra in Figure 12a and the map of full width at half maximum (FWHM) for the 2D mode in Figure S13a show that the process using Cu as a protective layer does not induce significant damage on graphene. Only a slight increase of the defect peak is observed after processing. The same can be observed in the sample using Ni as a protective stopping layer in Figure 12b and Figure S12b. Confirmation of the electrical contact is performed by acquiring transfer curves in electrolyte–gate configuration, as shown in Figure 12c,d. Both transfer curves exhibit the typical ambipolar behavior of EG-GFETs with the minimum conductivity point (V_CNP_) indicating intrinsic p-doping of the devices. We obtain a field-effect mobility for electrons and holes of μ_e_ ≈1700 cm^2^ V^−1^ s^−1^ and μ_h_ ≈1300 cm^2^ V^−1^ s^−1^, respectively, by fitting the transfer curves to a model describing the DC conductivity of single-layer graphene (SLG), σ, as a function of gate voltage. The model is based on carrier resonant scattering due to the strong short-range potentials originating from impurities adsorbed at the graphene surface [6,41].

### 3.3. Case Study: Fabrication at 200 mm Wafer Scale of Graphene Electrolyte Gated FETs

**Figure 13:**
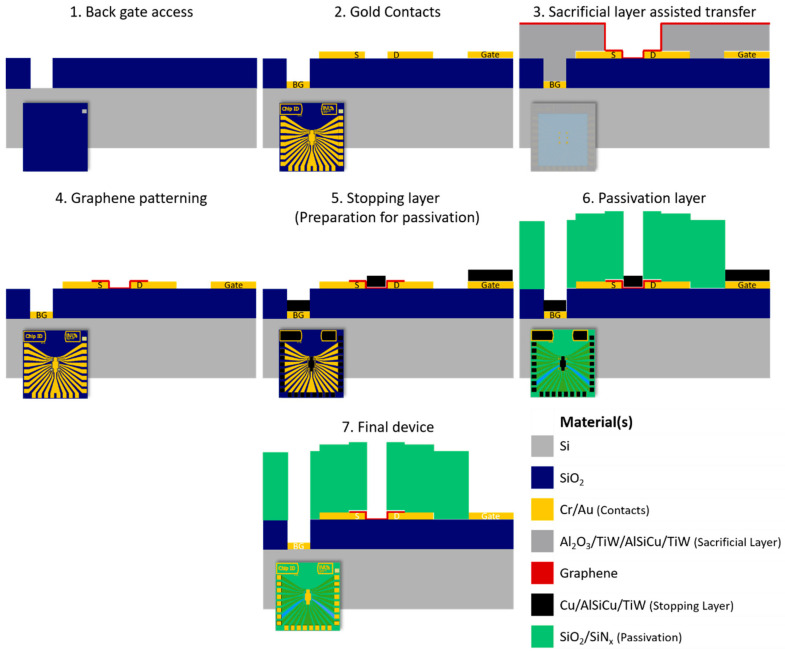
Graphical overview of the optimized fabrication process for wafer-scale production of EG-GFETs for biosensing. Steps 1 and 2 refer to contact patterning. In step 3, the sacrificial layer is used during graphene transfer to avoid transfer-borne residues on gold surfaces (e.g., gate electrode). After complete transfer and PMMA removal, graphene is patterned by dry etching, and the sacrificial layer is removed, as shown in step 4. In step 5, the stopping layer is prepared on top of graphene, based on Cu or Ni, which is followed by deposition and dry etch patterning of the passivation stack (step 6). Finally, the stopping layer is dissolved, and the final device is achieved (step 7). The color code for the layers/materials is presented at the bottom right corner of the figure.

This study’s main goal is to develop a process for the wafer-scale fabrication of high electronic quality devices for biosensing applications. The final devices produced at the 200 mm wafer scale following the process proposed in this paper are resistant to all the solvents used for graphene functionalization [16] and have low ion permeability, which increases their electrical stability. After the previous study, in which each step of the fabrication process is optimized using small area substrates, a complete microfabrication process is performed on a 200 mm Si wafer covered with 100 nm of thermal SiO2. The chip design is for 20 graphene transistors per chip, with a common source electrode for each group of 10 FETs, a top gate electrode in the center of each chip, and a back-gate access pad to allow double-gating of the transistors. The fabrication sequence is briefly presented in Figure 13.

The first step is to open the back-gate access using optical lithography and reactive ion etching (Figure S14a). Then, a contact layer stack is sputtered on the wafer: 3 nm of the Cr adhesion layer, 35 nm of the Au conductive layer, and 20 nm of the Al_2_O_3_ protective layer following the results of Section 3.1.4. The contacts are patterned using optical lithography and ion milling (Figure S14b) over 180 square dies, 18 dies of side 20.25 mm, and 162 dies of side 6.75 mm. Source and drain contacts are 75 μm wide (channel width, W), separated by a gap (channel length, L) of 25 μm. The gate electrode is circularly shaped with ≈20 mm2 area for the 20 mm dies and rectangular-shaped with ≈0.7 mm2 area for the 6.75 mm dies.

The sacrificial layer for graphene transfer is prepared by lift-off following Section 3.1.4, using TiW(N) in the top layer to ensure the sacrificial layer’s stability during the multiple graphene transfer steps. Optical microscopy is used to confirm the lift-off process’s completion (Figure S14c).

**Figure 14:**
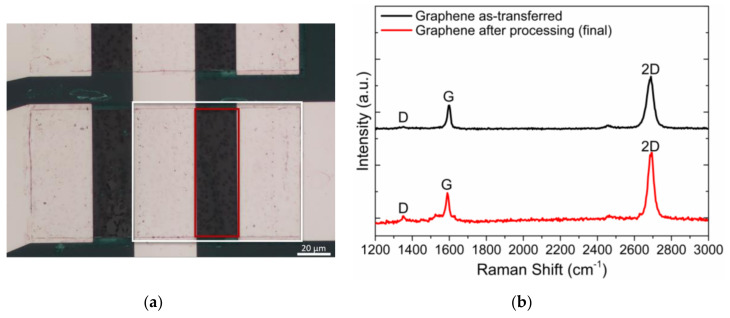
Graphene after transfer to 200 mm wafer and O_2_ plasma patterning. (**a**) Optical microscope photograph (50× magnification) showing the limits of the patterning and generally good coverage of graphene in the gold contacts (source and drain) and in the channel. The white rectangle limits the source and drain contacts of one EG-GFET, and the red region shows the channel area. The scale bar represents 20 μm. (**b**) Representative Raman spectra of graphene as transferred (black) and after completion of the fabrication process (red), showing the vibrational modes for defects (D), graphitic materials (G) and bi-dimensional characteristic of graphene (2D).

The processed PMMA/graphene films are transferred onto the wafer’s desired regions until full coverage is achieved (see Figure S14d and Figure 14 below). After PMMA removal, graphene is patterned with low power ECR-O2 plasma (Figure S14e and Section 3.1.1). After removing the sacrificial layer, graphene quality is accessed by optical microscopy (film continuity) and Raman spectroscopy for structural quality and continuity, as shown in Figure 14a,b.

Previously to the passivation, Al_2_O_3_ is selectively removed to improve adhesion of the passivation to the chips’ surface. Al_2_O_3_ is kept only at the gate and pads to prevent contact with the stopping layer by patterning 2200 nm thick AZP4110 photoresist and wet etching with alkaline solution (AZ400k 1:4). A hard coating for passivation is chosen (Section 3.2), considering the devices’ intended use. The process described in Section 3.2.2 is used to fabricate the passivation layer (Figure S14f,g) with a Cu or Ni-based stopping layer (black layer in Figure 13). The final wafer (Figure S14h) is analyzed with EDX to discard the presence of process contaminants (Figure S14i). Characterization using Raman spectroscopy was repeated after processing the wafer to confirm the achieved final quality of graphene (Figure 14b, red spectrum).

Optical images acquired after each fabrication stage (Figure S14) are used to monitor the process, ensuring that no wafer moves to the following fabrication step if the optical images show that the previous step is not successfully achieved. Raman spectroscopy of graphene as transferred and after processing shows a slight increase in defect concentration (D peak, 1350 cm^−1^). The defect-related D mode ratio to the G mode changes from 0.12 (as-transferred) to 0.24 (final), but 2D/G ≈ 2.3, indicating graphene quality, is not significantly affected by the fabrication process.

#### Statistical Study of the EG-GFET Channel Resistances Fabricated at the Wafer Scale

**Figure 15:**
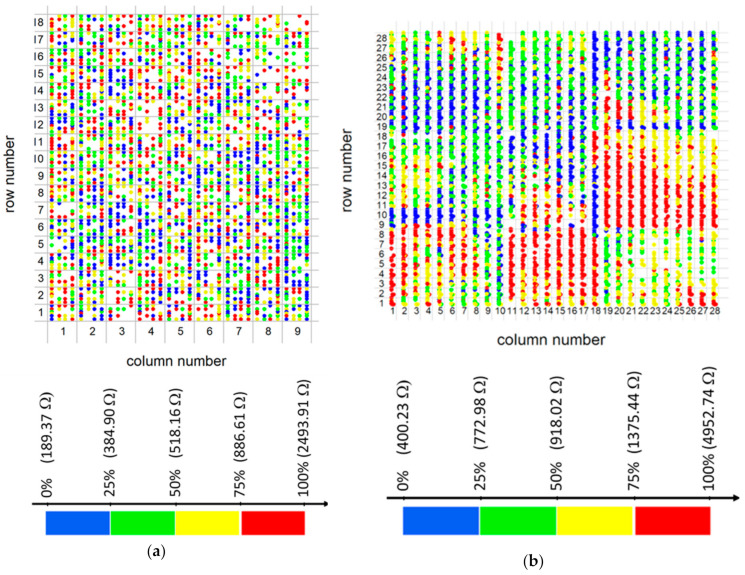

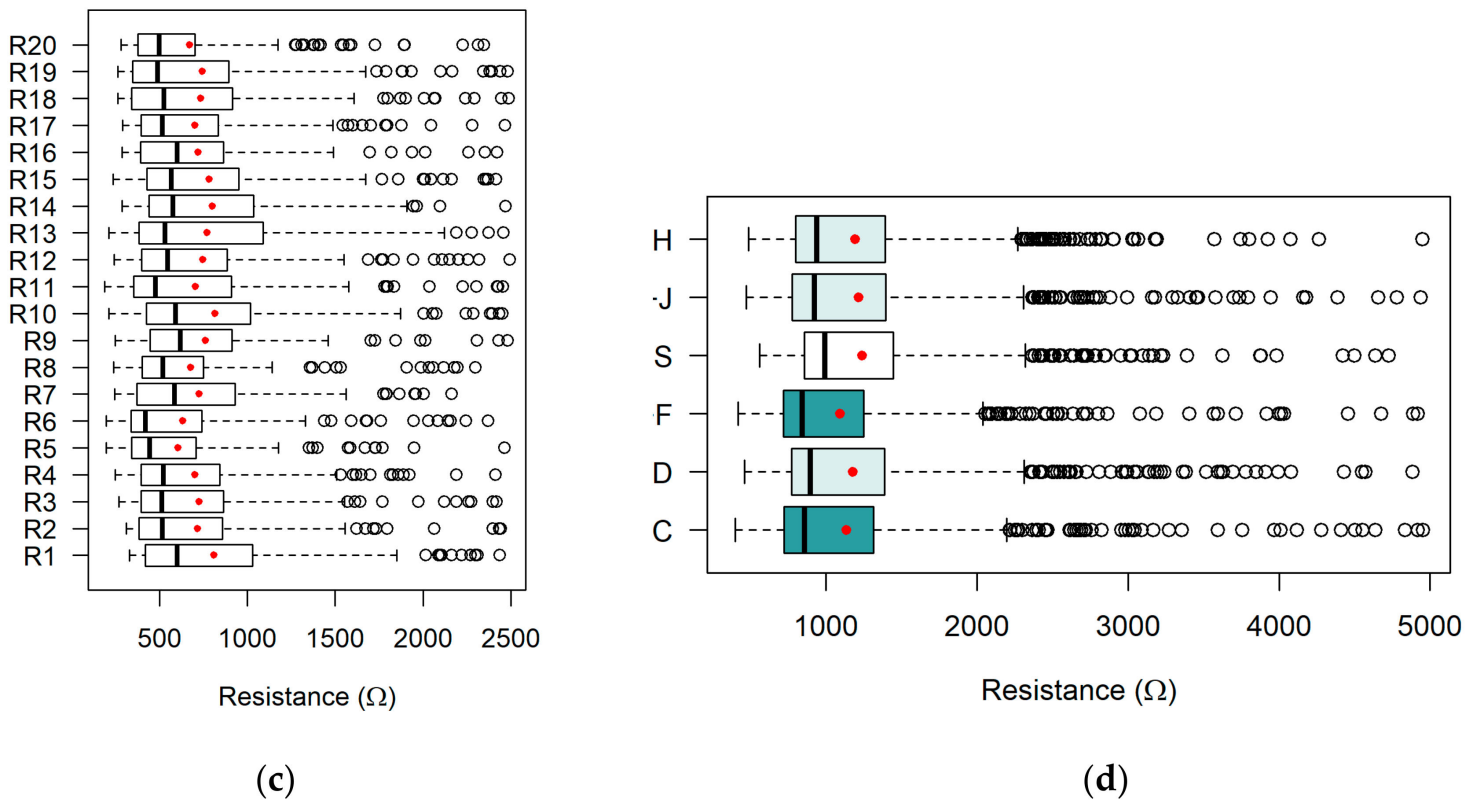
Analysis of resistance values distribution in the fabricated wafers. (**a**) Distribution of resistance values in wafer A, with color-coding according to the quartiles of R values’ empirical distribution. (**b**) Distribution of resistance values in wafer B, with color-coding according to the quartiles of R values’ empirical distribution. (**c**) Boxplot of the empirical distributions of resistance values per pair of contacts in wafer A. The black line inside each box represents the median, and the red dot represents the mean of each distribution. (**d**) Boxplot of the empirical distributions of resistance values per pair of contacts (A+C, B+D,… see text) in wafer B. The black line inside each box represents the median, and the red dot represents the mean of each distribution.

For the devices’ electrical characterization, an automated probe station is used to measure either the current or the voltage drop in the channel between source and drain contacts at a fixed voltage of 1 mV or a fixed current of 1 μA, respectively. Then, the measured values are converted to resistances to provide information on the homogeneity of the devices’ process and quality (Figure 15 and Figure S15a). For the geometry of the transistors (W/L = 3) and the unintentional doping level (see Figure 12c,d) of processed graphene, a channel resistance, *R*, below ≈1000 Ω is expected in successful devices, while values up to ≈2500 Ω are still acceptable. Transistors with *R* > 2500 Ω were rejected and discarded. Two wafers were processed and studied. Wafer A is a shared design wafer containing 9 × 18 chips (Figure S15b), each chip containing 20 transistors with resistances *R*_1_ to *R*_20_. In this wafer, measured at constant voltage, 63% of the 3240 channels have *R* < 1000 Ω (80% of the channels have R < 2500 Ω). In Figure 15a, each colored dot corresponds to one transistor’s location with *R* < 2500 Ω. The color code refers to four classes of data values according to quartiles, *q_i_* (*q*_0_ = 189.37 Ω, *q*_1/4_ = 384.90 Ω, *q*_1/2_ = 518.16 Ω, *q*_3/4_ = 886.61 Ω, *q*_1_ = 2493.91 Ω). The spatial distribution of the resistance values looks random, and no correlation with the graphene patches individually transferred (Figure S15c) is visible. The boxplot charts in Figure 15c display the data distributions per device (see device map in Figure S15d) showing that all contacts behave in a relatively similar way, with medians (averages, red dots) ranging from 421.12 Ω (606.45 Ω) for *R*_6_ (*R*_5_) to 617.96 Ω (816.83 Ω) for *R*_9_ (*R*_10_).

A second wafer (wafer B) was processed and measured at constant source–drain current. Figure 15b shows 4107 resistance measurements with values in the range from 400 Ω to 5000 Ω, belonging to 784 chips distributed across the wafer in a square array of 28 × 28 lines and columns. Each measurement encompasses two transistor channels connected in series. Hence, the acceptable resistance range was multiplied by two. Since six pairs of GFETs were measured per die (the remaining five pairs were not measured due to constraints imposed by the automated measuring system), we have 6 × 784 = 4704 measurements, of which those with resistance 2*R* > 5 kΩ were rejected (4704 − 4107 = 597). The x- and y-coordinates of each data point corresponding to one pair of resistors. The color code reflects four classes of data values according to quartiles, *q_i_* (*q*_0_ = 400.23 Ω, *q*_1/4_ = 772.98 Ω, *q*_1/2_ = 918.02 Ω, *q*_3/4_ = 1375.44 Ω, *q*_1_ = 4952.74 Ω). Figure 15d gives the data distributions per contact pair (A + C, B + D, E + F, G + S, I + J, J + H, see the chip contact map in Figure S15f), in boxplot format. The red dot inside each box represents the mean of each dataset. Medians (means) differ by contact pair, from 843 Ω (1095 Ω) for E + F to 993 Ω (1239 Ω) for G + S, revealing three categories of statistically different medians, as shown by different colors in the plot. The highest median category refers to G + S and the lowest refers to A + C and E + F, which are contacts located in the central part of the die. Variances do not differ statistically between contacts, whereas the empirical distribution tail is slightly longer for contact E + F and slightly shorter for G + S.

Figure 15b suggests the existence of nine regions across the wafer, according to the color code. In the fabrication sequence of wafer B, the graphene transfer process consisted of a succession of nine transfer steps of individual graphene patches, as arranged in Figure S15e. The correspondence of the nine zones between the figures is striking. This result shows that the classes of resistance values found in the measurements correlate with the graphene transfer step—a rather artisanal process—and less with any other fabrication steps.

## 4. Conclusions

A process for the fabrication of chips containing graphene electrolyte-gated FETs is developed step by step in this study, having in mind their application as biosensors. Many technical solutions for different process issues were suggested, developed, tested, and critically discussed. Some of them were rejected, given the study’s goal, since they do not provide chips with enough chemical or electrical stability. However, for less demanding applications, these solutions could be useful.

Dielectric passivation as the final step of the fabrication process allows an improvement of signal stability. It decreases electronic noise, since only the graphene channel and the gate electrode become exposed to the electrolyte during measurements. One of the most relevant results to obtain a useful chemical-resistant chip is the possibility of metal sputtering on top of graphene films without inducing relevant damage. This result can be extended to devices where metallic layers are required on top of graphene without significantly increasing the fabrication time.

A novel approach to improve the surface cleanliness of the chip surface after graphene transfer is demonstrated. An effective sacrificial layer is developed, allowing the patterning of graphene while protecting the pristine gold surfaces. This optimization is of high importance for using the devices as biosensors. It promotes better inter-device homogeneity and less random molecular adsorption responsible for the sensor signal’s random response and noise. The process is general and suitable for other applications or devices with similar requirements.

This study casts light on the critical field of microfabrication of graphene devices for chemical sensors with electric transduction and their chemical stability as a function of the process chosen in a manner that allows researchers in the field to pick, among the results presented, those that better suit their application.

## Figures and Tables

**Table 1:** List of materials used for microfabrication of EG-GFETs.

	Description	Supplier	Reference
**Materials**	Cu foil, 99.99 + % purity	Good Fellow, Huntingdon, UK	CU000410
	200 mm Si wafer, P-doped, 1–100 Ω cm, <100>	Silicon Valley Microelectronics, Inc., Santa Clara, CA, USA	0019808-006
	Si wafer with thermal oxide, 100 nm	Siegert Wafer, Aachen, Germany	8P0/1-100/725 ± 50/SSP/100 nm SiO_2_
	Hexamethyldisilazane (HMDS)	Technic, Saint-Denis, France	
	PMMA 15 kDa, powder	Sigma-Aldrich, St. Louis, MO, USA	1002366861
	PMMA 550 kDa, powder		43982
	FeCl3, 98% purity	Alfa Aesar, Haverhill, MA, USA	12357
	KOH flakes, 90% purity	Sigma-Aldrich, St. Louis, MO, USA	484016
**Solvents**	Dimethylformamide (DMF) 99.75% purity	Sigma-Aldrich, St. Louis, MO, USA	227056
	Acetone, 99.5% purity	Honeywell, Charlotte, NC, USA	606-001-00-8
	Isopropanol (IPA), 99.8% purity	Honeywell, Charlotte, NC, USA	603-117-00-0
	Anisole, ≥99% purity	Sigma-Aldrich, St. Louis, MO, USA	801452
**Photoresists**	AZ1505	Microchemicals GmbH, Ulm, Germany	10052110018
	AZP4110	Microchemicals GmbH, Ulm, Germany	18451023159
	mrDWL1_XP	Micro Resist Technology, Berlin, Germany	UN1760
**Developers**	AZ400k 1:4	Microchemicals GmbH, Ulm, Germany	10063823163
	AR 300.47	Allresist, Strausberg, Germany	1912328
	mrDev600	Micro Resist, Berlin, Germany	R815100
**Solutions**	HCl 37%	Sigma-Aldrich, St. Louis, MO, USA	320331

## Supplementary Materials

The following are available online at https://www.mdpi.com/1996-1944/13/24/5728/s1, Figure S1: Raman spectra of graphene as grown in Cu foil (a) and after transfer to a SiO_2_ substrate with gold contacts (b). Figure S2: Optical microscopy photographs of a chip surface before and after graphene transfer. Figure S3: Photoresist profiles after exposure to different times of low-power O2 plasma. Figure S4: Optical Microscope images of sample surface before graphene patterning and after patterning. Figure S5: Optical microscope images of samples with pre-patterned gold contacts after graphene transfer/patterning assisted by lift-off. Figure S6: Proposal (a) and execution (b) of the modified lift-off strategy for graphene transfer and patterning in two steps. Figure S7: Graphene transfer and patterning using a protective sacrificial layer. Figure S8: Sacrificial layer damage induced by HCl residues of graphene transfer process. Figure S9: Film thickness after different etching times for SiNx and SiO_2_. Figure S10: Sample after wet etch in KOH 1M at 60 °C. Figure S11: Raman spectra of graphene samples before and after sputtering and wet etch. Figure S12: EDX spectra at the gate electrode after patterning of the dielectric passivation and after each step of the wet etch. Figure S13: Full width at half maximum (FWHM) of the 2D mode of graphene after transfer and patterning by stopping layer-assisted patterning of the passivation. Figure S14: Optical microscope photographs resuming the steps of the wafer-scale fabrication process of EG-GFETs. Figure S15: Complementary information of the electrical characterization of wafers A and B.
