# Supplementary material for: Clean-Room Lithographical Processes for the Fabrication of Graphene Biosensors
Source: Materials (Basel). 2020 Dec 15;13(24):5728. doi: 10.3390/ma13245728 (PMC7765539; doi:10.3390/ma13245728)
Supplement: Supplementary file 1 [file materials-13-05728-s001.zip › materials-968548-supplementary.pdf]

materials

Supplementary Material

Clean-Room Lithographical Processes for the Fabrication of Graphene Biosensors

Patrícia D. Cabral [1,2], Telma Domingues [1], George Machado Jr. [1], Alexandre Chicharo [1], Fátima Cerqueira [1,2], Elisabete Fernandes [1], Emília Athayde [3], Pedro Alpuim [1,2,*] and Jérôme Borme [1]

1 International Iberian Nanotechnology Laboratory, 4715-330 Braga, Portugal; patricia.silva@inl.int (P.D.C.); telma.domingues@inl.int (T.D.); george.junior@inl.int (G.M.J.); alexandre.chicharo@inl.int (A.C.); fatima.cerqueira@inl.int (M.F.C.); elisabete.fernandes@inl.int (E.F.); jerome.borme@inl.int (J.B.)
2 Center of Physics, University of Minho, 4710-057 Braga, Portugal
3 Center of Mathematics, University of Minho, 4710-057 Braga, Portugal; mefqa@math.uminho.pt
* Correspondence: pedro.alpuim.us@inl.int;

S1. Materials and Methods

S1.1 Clean-Room Fabrication Tools

S1.1.1. Deposition Tools

For thin film deposition on the 200 mm wafer and 4 × 4 cm samples, different sputtering and chemical vapor deposition (CVD) tools are used depending on the material requirements. A Kenosistec DC magnetron sputtering system is used to deposit thin metallic films of Cr, Au (contacts), Cu, and Ni (stopping layer). The base sputtering pressure is between 5×10^{-7} and 8×10^{-7} mbar. The sputtering conditions are 20sccm Ar, 80 W, 300 mA for Cr; 6 sccm Ar, 100 W, 200 mA for Au; 20 sccm Ar, 100 W, 100 mA for Cu; and 15 sccm Ar, 100 W, 300 mA for Ni. A Timaris FTM RF sputtering system is used to deposit films of Al_2O_3 (RF cathode, 200 sccm Ar, 2570 W), TiW(N) (DC cathode, 200 sccm Ar, 40 sccm N, 1000 W, 3 A) and AlSiCu (DC cathode, 200 sccm Ar, 2250 W, 6 A) for the sacrificial and stopping layers. All sputtered materials are obtained from high purity sputter targets. The AlSiCu mass composition is $Al_{98.5}Si_1Cu_{0.5}$ (mass fraction). TiW(N) is obtained from $Ti_{10}W_{90}$ (mass fraction) and sputtered in N plasma.

An SPTS MPX plasma-enhanced CVD, Newport, United Kingdom, is used to deposit films of SiO_2 and Si_xN_y for the passivation layer.

For graphene growth, a FirstNano EasyTube® 3000 thermal CVD system is used as described in the section S1.2.3.

S1.1.2. Lithographic Tools

To prepare the wafer/samples for lithography, HMDS silanization is performed with YES Vapor Prime Oven 310TA, Livermore, CA, USA, to improve photoresist adhesion. Coating with the photoresists AZ1505 and AZP4110 is performed with Suss Microtec Gamma Cluster tracks, Garching bei München, Germany, with baking temperatures of 100 °C and 110 °C, respectively. Patterning of the photoresist films is done using Direct Write Laser Lithography—DWL2000 with 405 nm laser. The masks for patterning are prepared with LibreCAD software.

S1.1.3. Dry Etch Tools

The tools for dry etching are divided into two groups: the patterning of thin films and patterning of graphene. For patterning of the contact layer, a Nordiko Ion Milling system, Havent, UK, is used, with 300 s of etching at an angle of 40° between the wafer normal and the incident ionic stream. The Ar plasma is 440 W with a working pressure of 4×10^{-4} Torr, and the ions are accelerated by a −3200 V grid before neutralization.

For patterning of the passivation layer, an SPTS APS SiO_2 Etcher, Newport, UK, is used with 150 s of plasma, for complete etching of the passivation, including 25% overetch. The reactive mixture contained C_4F_8 50 sccm, H_2 30 sccm, and the working pressure is 6×10^{-3} mbar.

High-power O_2 plasma in a Roth & Rau cold wall plasma-enhanced CVD system is used to clean the back side of the graphene catalyst Cu foil for 4 min (8 cycles of 30 s plasma) under 200 W of plasma and pressure 4×10^{-1} mbar.

For patterning graphene, low-power O_2 plasma is used in a PVA TEPLA Plasma Asher, Wettenberg, Germany, for 18 min (3 cycles of 6 min plasma) in conditions: 230 W, O_2:Ar 1:1, 0.5 mbar, or high-power O_2 plasma is used in a SPTS ICP system for 30 s in conditions: 1200 W, O_2 100 sccm, 3×10^{-2} mbar.

S1.1.4. Dicing Tools

To cut 4 cm by 4 cm testing samples and to dice the wafer into individual chips, a Disco DAD 3500 Automatic Dicing Saw, Tokyo, Japan, is used, with a Mikrokerf 2.187-10-30H (hub-type blade, 254 μm width, 30 μm diamond grit in hard resin). Previously to dicing, the wafers are coated with AZ1505 photoresist to keep the devices free of debris. The blade water flux is reduced to half the usual value to prevent damage to the graphene surfaces. The blade is spinning at 30 krpm, and the feed speed is 8 mm/s.

S1.2. Microfabrication Methods

S1.2.1. Dry Etch Patterning

For dry etch patterning, the samples were coated with positive photoresist, AZ1505, and the pattern was written using a DWL2000 optical lithography system with a CAD mask and 405 nm laser. After exposure, the samples were developed with AZ400k 1:4 developer for pre-determined times according to the film thickness. Dry etch was performed using different equipment, according to the etched layer.

S1.2.2. Fabrication of Metal Contacts and Interconnects

Following the strategy of maximizing lithography process steps before graphene transfer, we start the fabrication of the EG-GFET by depositing and patterning the source, drain, and gate contacts. A 200 mm Si wafer with 100 nm of thermal oxide is coated by sputtering with an adhesion layer of chromium (Cr, 3 nm) followed by a conductive layer of gold (Au, 35 nm) and a sacrificial layer of alumina (Al_2O_3, 10 nm). The contacts are patterned into 513 square dies of 6.75 mm side using optical lithography and ion milling to etch the unprotected Cr/Au/Al2O3 into the desired pattern. The etching process is followed by Secondary Ion Mass Spectroscopy (SIMS) using as end-point criterion the extinction of the Cr signal. The gap between source and drain contacts (the channel length) is fixed at 25 μm, and the width of the gold contacts is 83 μm in all devices (the channel width is 75 μm). The layout of the contacts is made to accommodate 20 graphene EG-GFETs divided into 4 groups of 4 FETs and 2 groups of 2 FETs, which is a layout specifically developed to perform multiplex assays in biosensing. To perform the tests described in the following sections, the Si wafer is diced in chips of 4×4 cm^2, each containing 36 dies.

S1.2.3. Graphene Growth and Transfer

Single-layer graphene (SLG) was grown by chemical vapor deposition in a load-locked quartz tube 3-zone furnace (FirstNano EasyTube® 3000) onto 99.99 + % purity copper (Good Fellow) foils (25 µm thickness). A gaseous mixture of methane/hydrogen at a gas flow rate ratio of CH_4:H_2 1:60 was used for growth. Graphene grows on both sides of the copper foil. For graphene transfer, a temporary poly(methyl methacrylate) (PMMA) substrate was used. PMMA was spin coated onto the top side of the graphene/Cu/graphene sample and copper was further dissolved by dipping the PMMA/graphene/Cu into a 0.5 M $FeCl_3$ solution for 2 h. PMMA/graphene was cleaned in 2% HCl solution to remove metal precipitates and further washed in deionized water three times. PMMA/graphene films were transferred to the pre-patterned silicon/silicon dioxide (Si/SiO_2) wafer substrate. After transfer, the sample is dried in air overnight. Then, the PMMA is removed using acetone. Graphene quality, i.e., the homogeneity of the obtained graphene film after transfer, is first investigated by optical images. Confocal Raman spectroscopy was used to confirm the presence of SLG.

S2. Results and Discussion

S2.1. How to Transfer and Pattern Graphene Leaving a Clean Wafer Surface

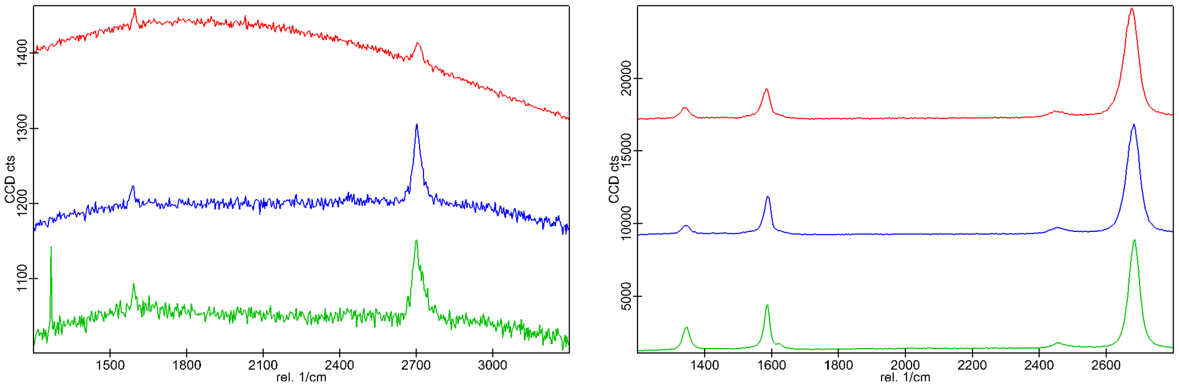

(**a**) (**b**)

Figure S1. Raman spectra of graphene as grown in Cu foil (**a**) and after transfer to an SiO_2 substrate with gold contacts (**b**) in three different sample points (red, blue and green represent individual sample points).

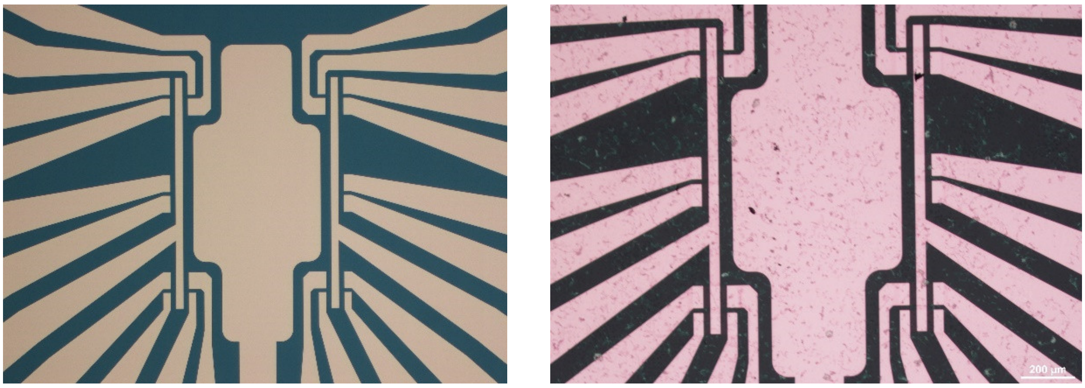

(**a**) (**b**)

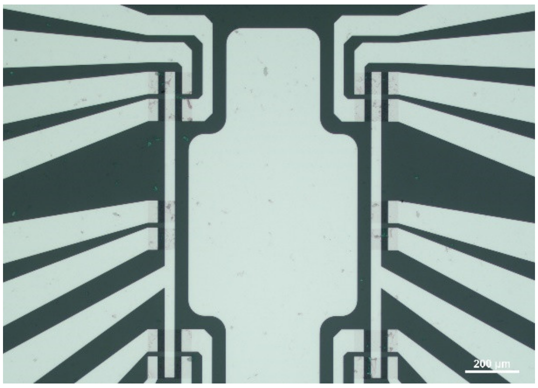

(**c**)

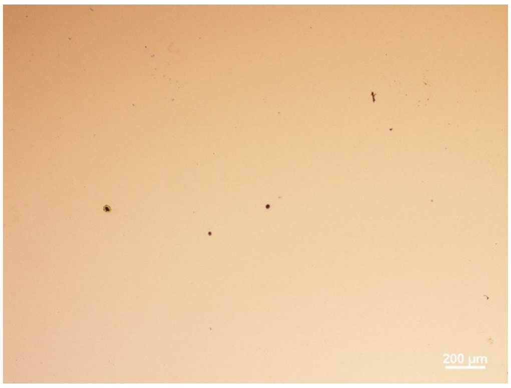

(**d**)

Figure S2. Optical microscopy photographs of a chip surface before and after graphene transfer. (**a**) Pristine surface with no visible contaminants with 5× magnification. (**b**) After graphene transfer, a great number of small particles are visible, even at 5× magnification. (**c**) After graphene removal through O_2 plasma processes, the particles/contaminants are still visible at 5× magnification, meaning they are permanently attached to the surfaces. (**d**) Transfer-borne residues on gold surface after graphene removal at 5× magnification. Scale bar of 200 µm for all photographs.

S2.1.1. Dry Etching

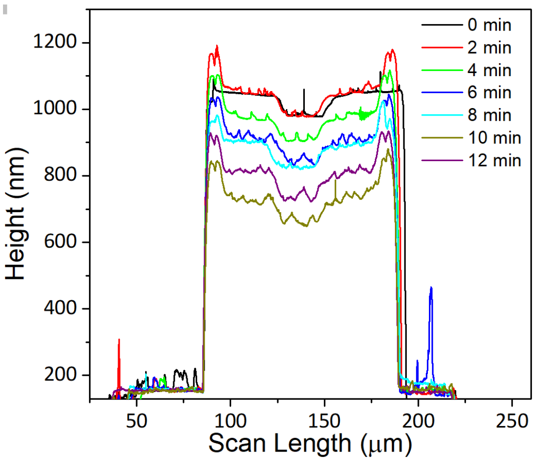

(**a**)

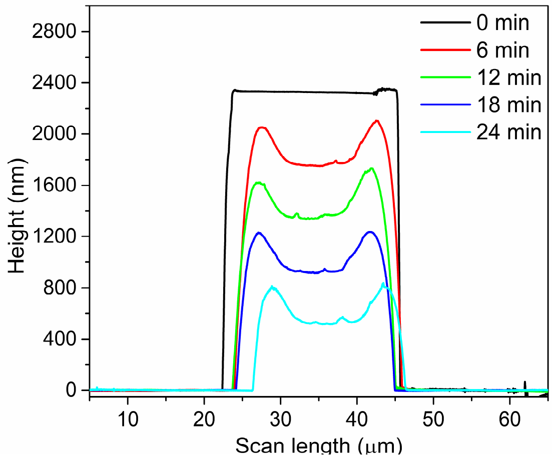

(**b**)

Figure S3. Photoresist profiles after exposure to different times of low-power O_2 plasma. (**a**) Profile of AZ1505 photoresist after each 2 min of O_2 plasma, with an initial thickness of ≈1 µm, showing decrease in photoresist thickness in each additional 2 min step. (**b**) Profile of AZP4110 photoresist after each 6 min of O_2 plasma, with initial thickness of ≈2.4 µm, showing a decrease in photoresist thickness in each additional 6 min step. All profiles were obtained with KLA—Tencor P-16 Surface Profiler.

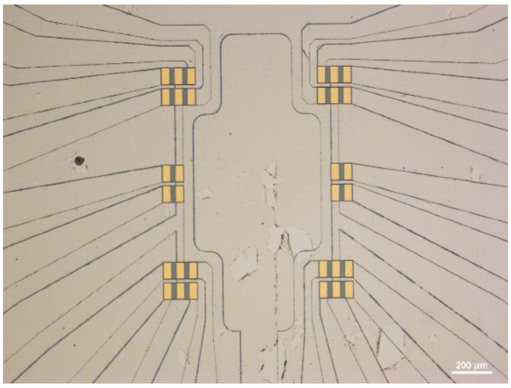

(**a**)

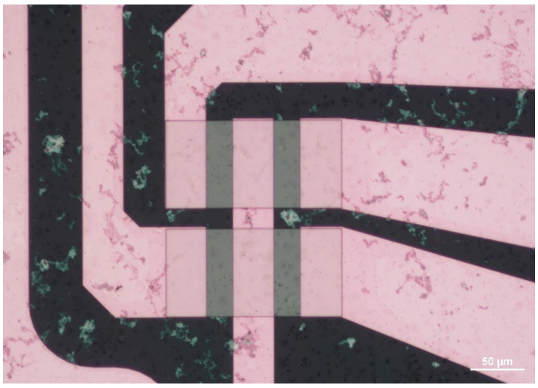

(**b**)

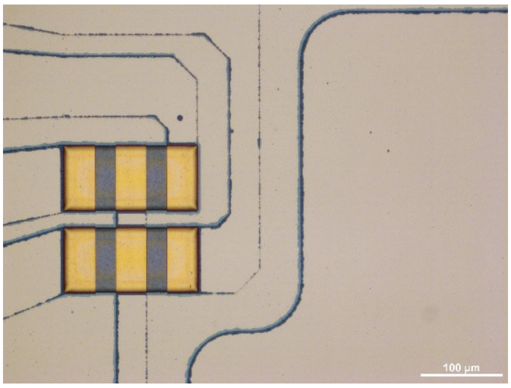

(**c**)

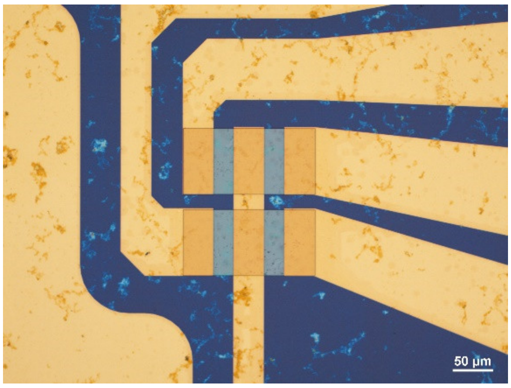

(**d**)

Figure S4. Optical microscope images of sample surface before graphene patterning (**a**,**b**) and after patterning with electron cyclotron resonance (ECR) (low power) O_2 plasma source (**c**) and inductively-coupled (ICP) (high power) O_2 plasma source (**d**). Scale bars represent 200 µm for (**a**), 100 µm for (**c**), and 50 µm for (**b**) and (d).

S2.1.2. Lift-Off Based Transfer

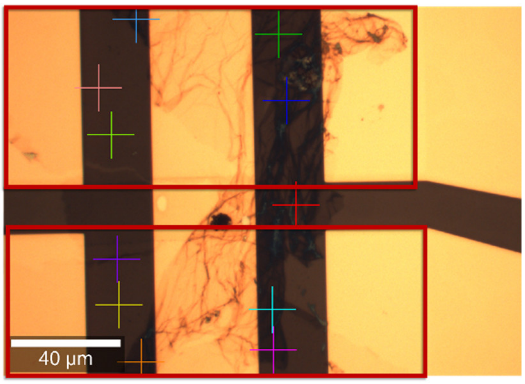

(**a**)

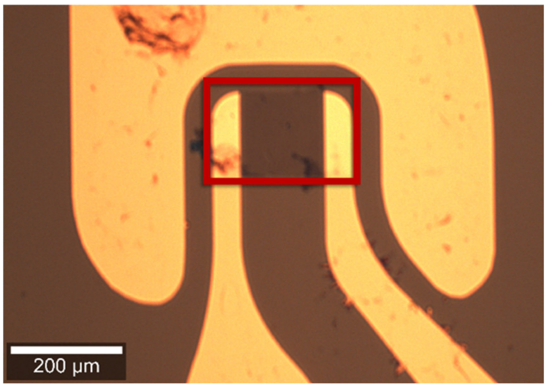

(**b**)

Figure S5. Optical microscope images of samples with pre-patterned gold contacts after graphene transfer/patterning assisted by lift-off. The size and density of the structures influences the efficiency in controlling the breaking step, with small features (**a**) becoming poorly patterned and big features (**b**) becoming well defineS2.1.3. Combined Approach

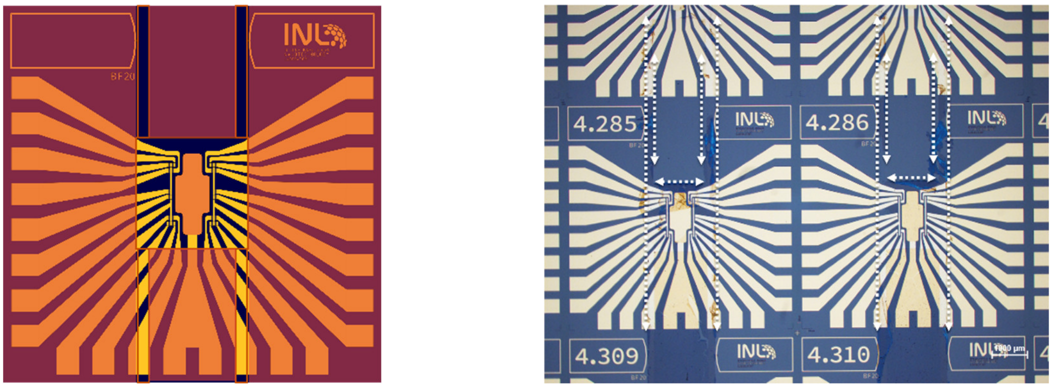

(**a**) (**b**)

Figure S6. Proposal (**a**) and execution (**b**) of the modified lift-off strategy for graphene transfer and patterning in two steps. Only the result of the first step (lift-off assisted transfer) is shown in (b), since the goal of gate protection is not achieved. The white arrows show the regions were graphene cracked in the mask limits. The darker region on the gate of device 4.285 is evidence of the re-deposition of loose graphene pieces during the lift-off process. In the device 4.286, it is also visible that graphene did not break along the gate features, which were also protected with photoresist prior to the transfer.

S2.1.4. Pre-Transfer Sacrificial Layer

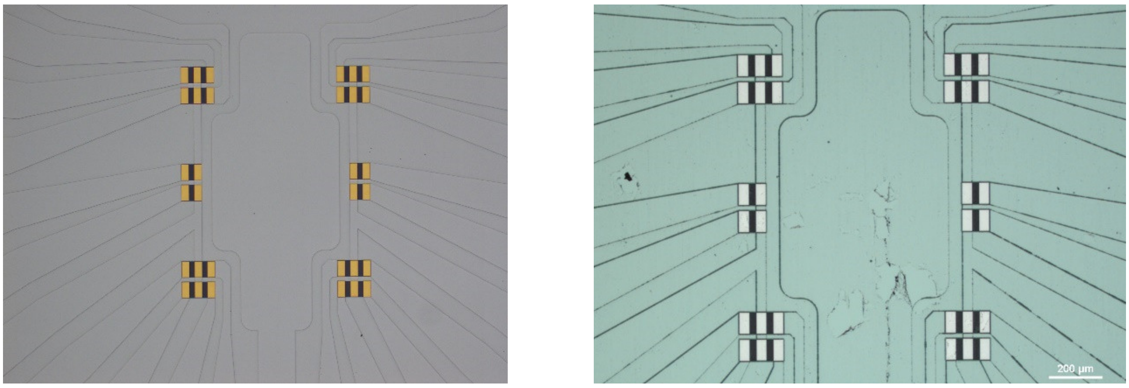

(**a**) (**b**)

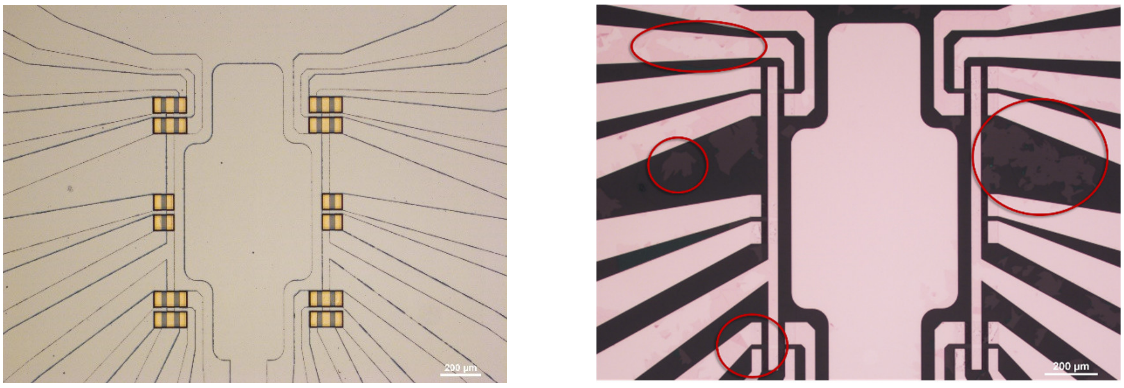

(**c**) (**d**)

Figure S7. Graphene transfer and patterning using a protective sacrificial layer. After preparing the sacrificial layer by lift-off (**a**), graphene was transferred (**b**) and patterned using O_2 plasma (**c**). The sacrificial layer was

removed (**d**) by wet etch while keeping the patterning photoresist on the sample. Scale bar of 200 μm for all photographs.

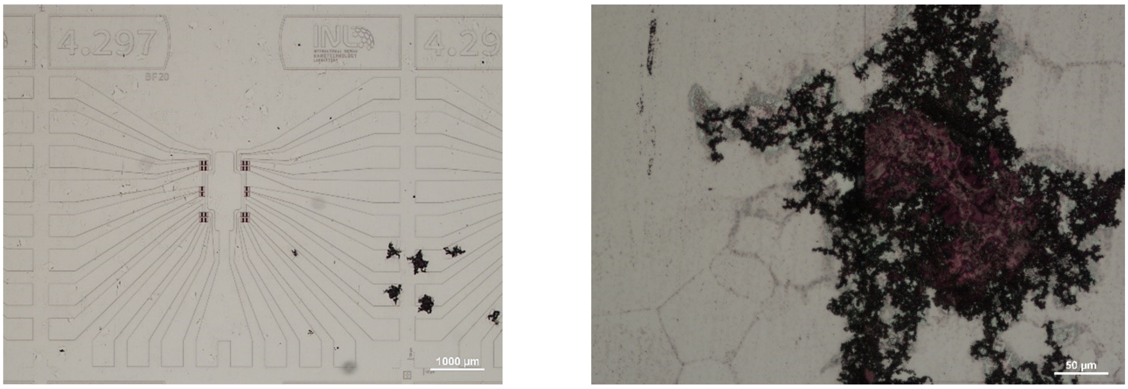

(**a**) (**b**)

Figure S8. Sacrificial layer damage induced by HCl residues of the graphene transfer process. (**a**) Damage occurs in small areas, as observed by the darker coloring of the sacrificial layer. Scale bar represents 1000 μm. (**b**) Inset of one of the damaged regions, exhibiting the TiW(N) layer beneath the "attacked" AlSiCu. Scale bar represents 50 μm.

S2.2. Fabrication of the Dielectric Passivation Layer

S2.2.1. Combined Reactive Ion Etching and Wet Etch Strategy

Two test wafers are prepared to measure the dry and wet etch rate of the passivation layer, as follows: Si wafers are coated, using PECVD, with 90 nm of SiO_2 and 50 nm of SiN_x on Test Wafer #1, and 120 nm of SiO_2 and 50 nm of SiN_x on Test Wafer #2. Based on previous etching recipe parameters, historical values of etch rates for SiO_2 (116 nm/min) and SiN_x (274 nm/min) are used to estimate the time required to completely etch the top SiN_x layer and about half of the SiO_2 layer. Test Wafer #1, is etched over times ranging from 20 to 30 s. After performing reactive ion etching (RIE), an interferometer is used to measure the remaining thickness of the SiO_2 film in the pattern. The estimated thicknesses are presented as a function of etch time in Figure 3a. Due to the fast decrease of the SiO_2 thickness, shown in Figure 3a (black squares), which increases the error in the estimation of the post-etch film thickness, a new wafer is prepared (Test Wafer #2) with an increased thickness of the SiO_2 layer. For this thicker film, after 25 to 30 s of etch time (Figure 3), red dots) approximately 40 nm of SiO_2 is left on top of the hypothetic graphene, thus preventing damage to the 2D film.

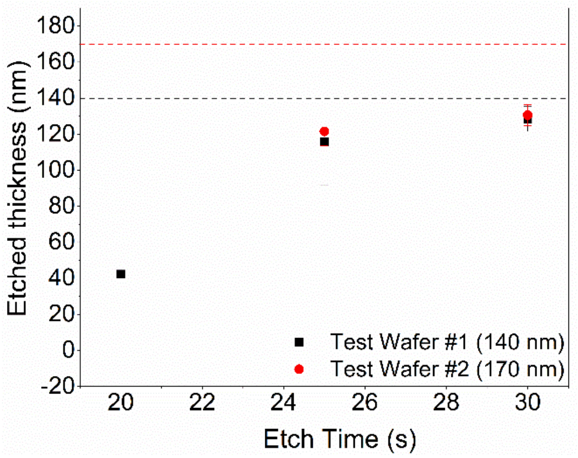

(**a**)

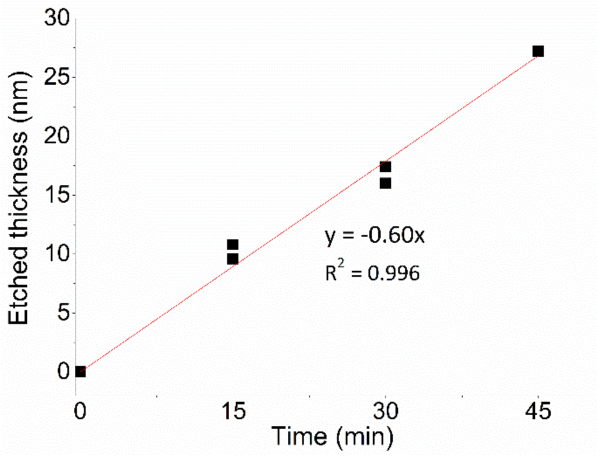

(**b**)

Figure S9. Film thickness after different etching times for SiN_x and SiO_2. (a) Dry etch of SiN_x and SiO_2 stack for two different initial thicknesses. The dashed lines refer to the reference thickness for each test wafer. (b) Wet etch of SiO_2 films by KOH 1M at 60 °C overtime.

The second step of the dielectric passivation—wet etch—is tested using features previously patterned by RIE. The Test Wafers #1 and #2 are diced in samples of 2 × 2 cm^2 and exposed to KOH 1M for different times and temperatures until a satisfactory etching rate is obtained. When exposed to KOH 1 M at 45 °C, the etch rate is about 5 nm/h, which is similar to the room temperature rate [31]. Increasing the temperature to 60 °C leads to a 7-fold increase in etch rate, to approximately 36 nm/h. The etch rate is estimated by exposing several samples to KOH for 15 min, 30 min, or 45 min. The thickness of the SiO_2 film is measured using the interferometer, before and after etching, and the difference is plotted as a function of time. A least squares fit estimates the etch rate, as shown in Figure 3b.

Once both steps of the passivation layer are optimized on featureless unpatterned films a sample with patterned contacts (as described in Section 3.1) is prepared to undergo the same procedure, mimicking the complete process to be performed at wafer scale. After graphene transfer and patterning, the passivation layer (120 nm of SiO_2 and 50 nm of SiN_x) is deposited, and lithography is performed to expose the channel and gate areas to the RIE. Since the features are too small for thickness estimation by interferometry, AFM is used to measure the remaining thickness of SiO2 after RIE and estimate the time required for the subsequent wet etch step. The data from the AFM measurement, as shown in Figure S1, gives a step of approximately 100 nm, i.e., there is still 70 nm of SiO_2 left on top of the graphene channel. Based on the wet etch study performed in the test wafers, the sample is immersed for 2 h in the KOH 1 M etching solution at 60 °C. However, after this process, severe peeling-off of the passivation layer is noticeable even to the naked eye, as shown in Figure S10.

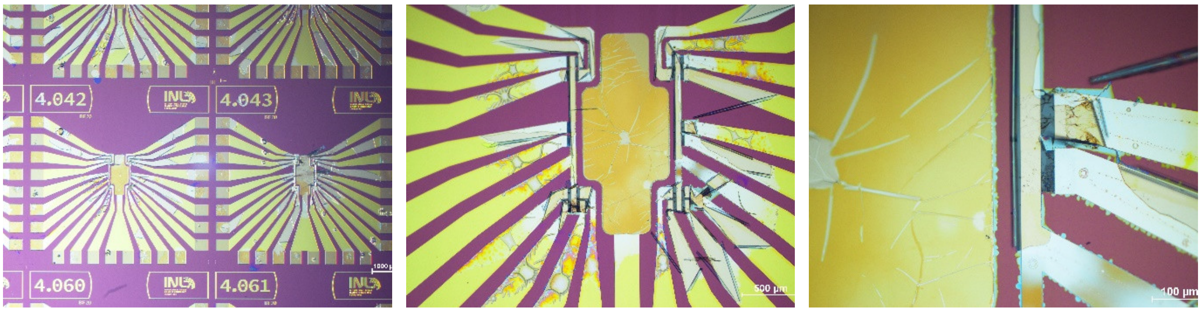

(**a**) (**b**) (**c**)

Figure S10. Sample after wet etch in KOH 1M at 60 °C. A peeling effect is very clear even at 1× magnification (**a**). Increasing the magnification to 5×, it is noticeable that the peeling is starting at the channels region, which is confirmed at 20× magnification (**c**), indicating that the peeling is caused by an undercut of the SiO_2 during wet etch with KOH. Scale bars represent 1000 µm (**a**), 500 µm (**b**), and 100 µm (**c**), respectively.

S2.2.2. Stopping Layer Assisted RIE of Dielectric Passivation

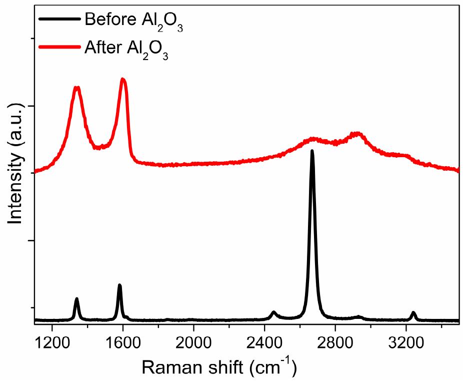

(**a**)

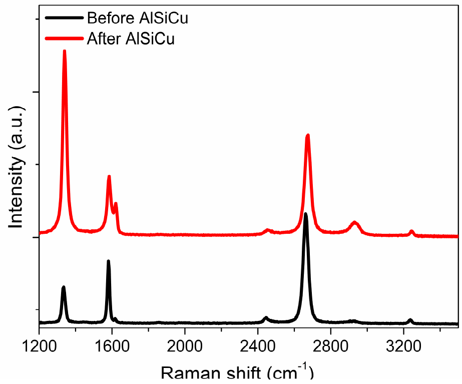

(**b**)

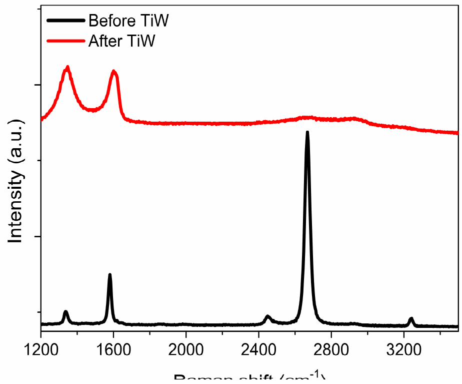

(**c**)

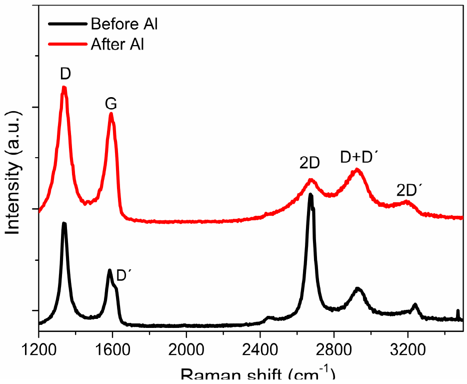

(**d**)

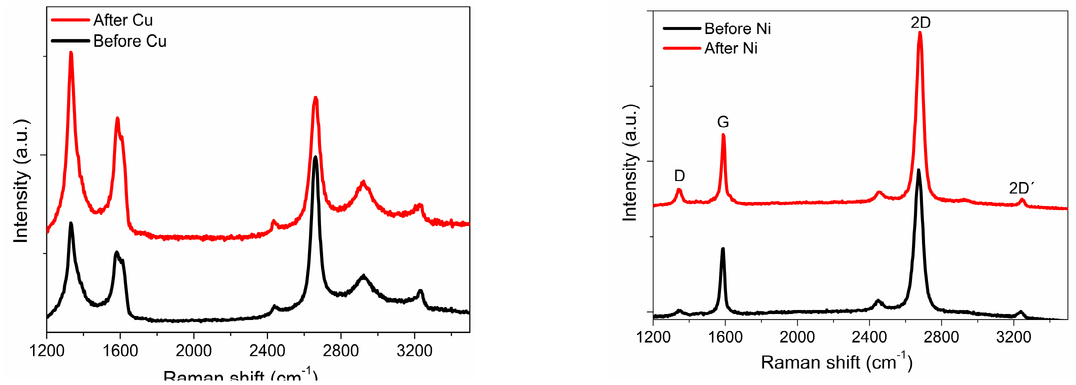

(**e**) (**f**)

Figure S11. Raman spectra of graphene samples before and after sputtering and wet etch of Al_2O_3 (**a**), AlSiCu (**b**), TiW(N) (**c**), Al (**d**), Cu (**e**), and Ni (**f**).

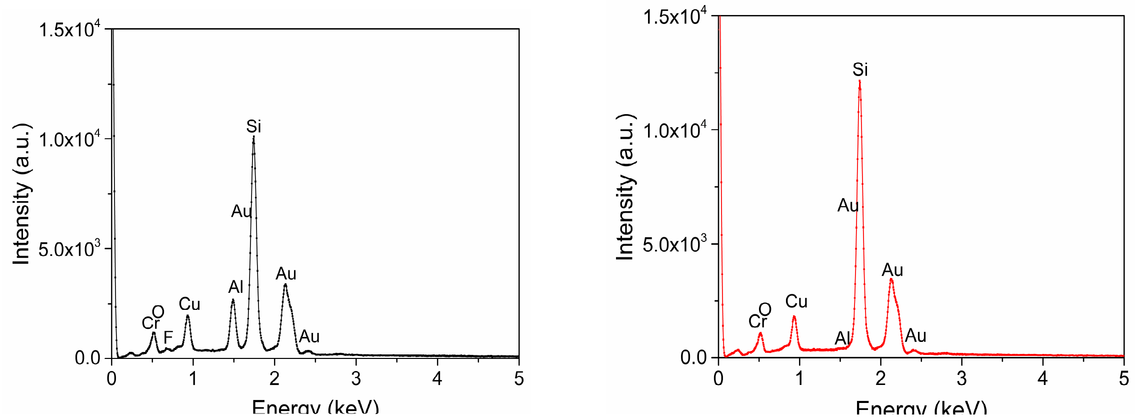

(**a**) (**b**)

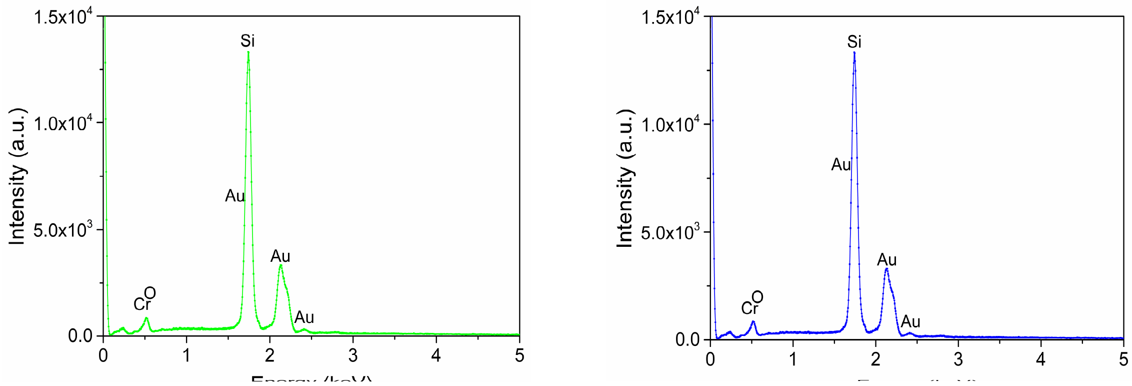

(**c**) (**d**)

Figure S12. Energy-dispersive X-ray spectroscopy (EDX) spectra at the gate electrode after patterning of the dielectric passivation (**a**) and after each step of the wet etch. (**b**) After AlSiCu removal by AZ400k 1:4. (**c**) After Cu removal by $FeCl_3$ 0.5 M. (**d**) After Al_2O_3 removal by AZ400k 1:4.

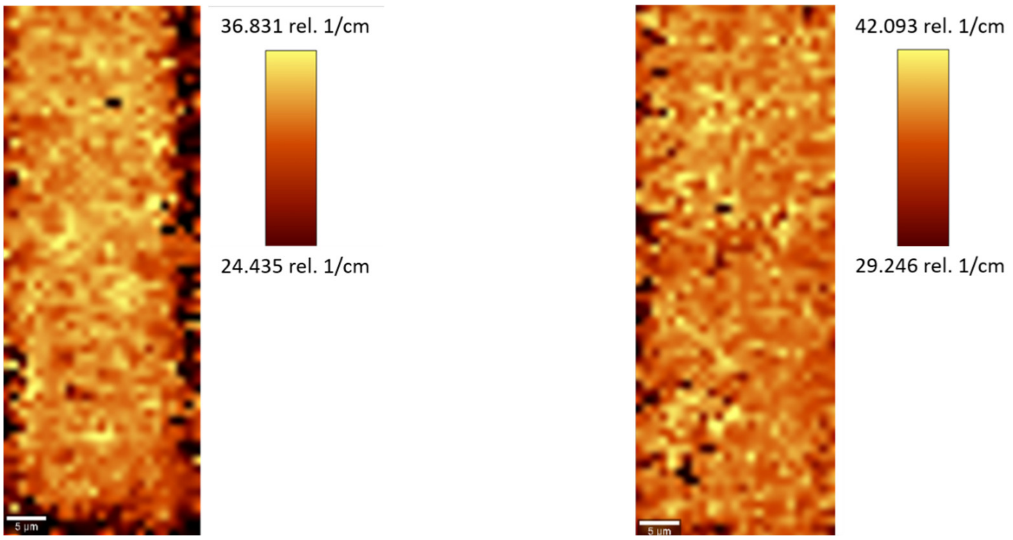

(**a**) (**b**)

Figure S13. Full width at half maximum (FWHM) of the 2D mode of graphene after transfer and patterning by stopping the layer-assisted patterning of the passivation. (**a**) FWHM for a graphene channel after process using a Cu-based stopping layer. (**b**) FWHM for a graphene channel after the process using an Ni-based stopping layer.

S2.3. Case study: fabrication at 200 mm wafer scale of graphene electrolyte gated FETs

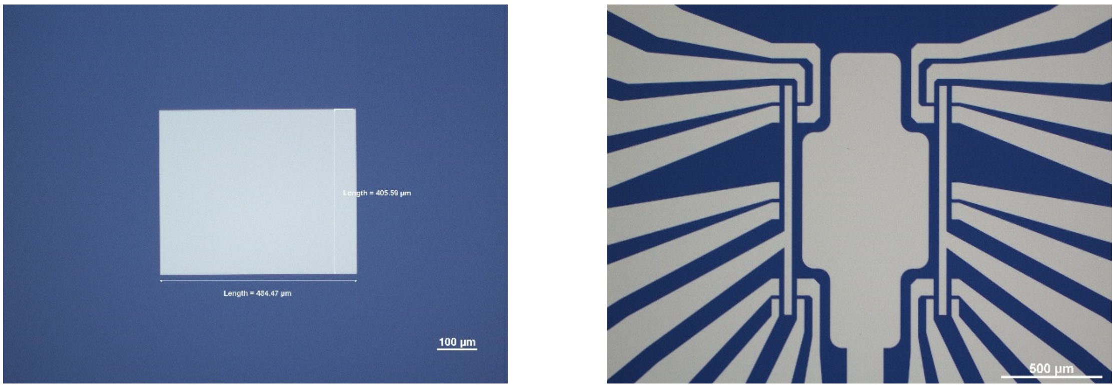

(**a**) (**b**)

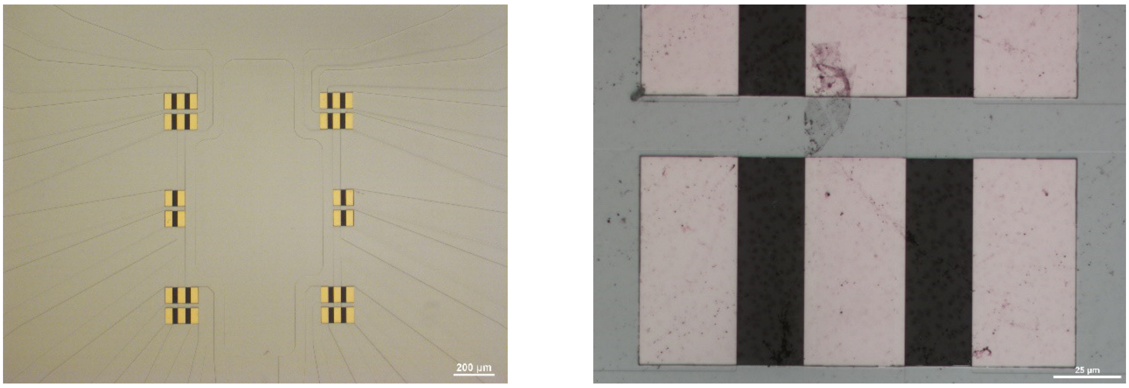

(**c**) (**d**)

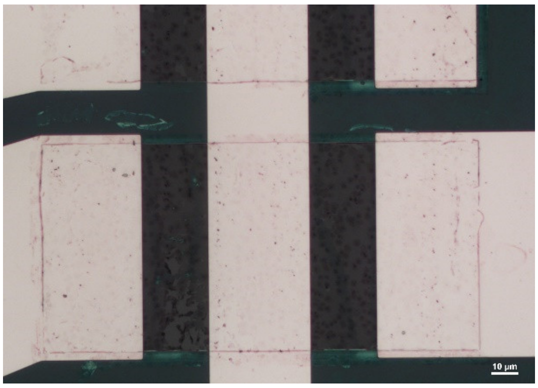

(**e**)

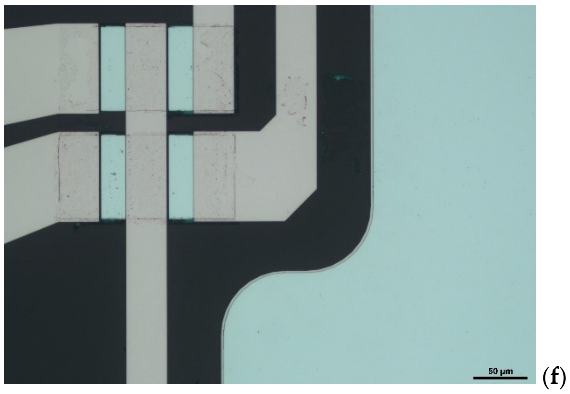

(**f**)

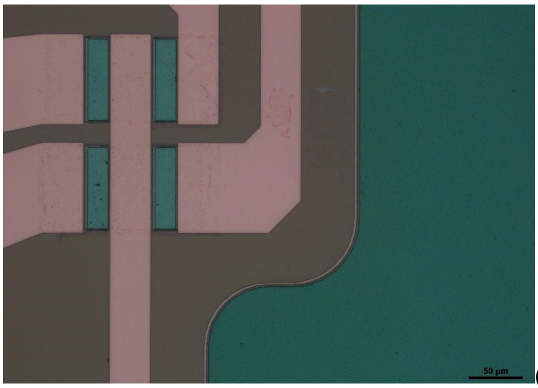

(**g**)

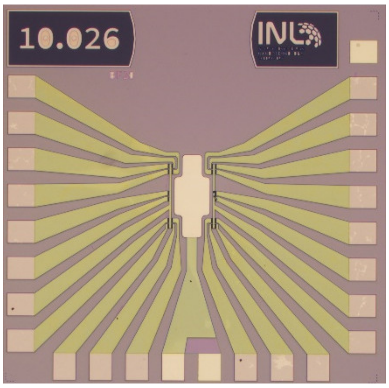

(**h**)

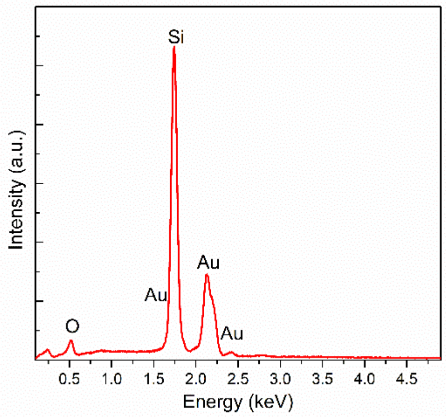

(**i**)

Figure S14. Optical microscope photographs resuming the steps of the wafer-scale fabrication process of graphene field-effect transistors in which liquid electrolyte replaces the commonly used solid dielectrics (EG-GFETs). (**a**) Back gate access opened by reactive ion etching (RIE) (20× magnification). Scale bar represents 100 µm. (**b**) Patterned Au contacts for 20 EG-GFETs with one common in-plane gate electrode (5× magnification). Scale bar represents 500 µm. (**c**) Sacrificial layer for graphene transfer composed of TiW(N), AlSiCu, and TiW(N) (5× magnification). Scale bar represents 200 µm. (**d**) Surface of the wafer after graphene transfer (50× magnification). Scale bar represents 25 µm. (**e**) Same area after patterning by O_2 plasma and removal of the sacrificial layer (50× magnification). Scale bar represents 10 µm. (**f**) After preparation of the stopping layer composed by Ni, AlSiCu, and TiW(N) (20× magnification). Scale bar represents 50 µm. (**g**) After deposition of the dielectric passivation stack (20× magnification). Scale bar represents 50 µm. (**h**) After RIE patterning of the passivation and removal of the stopping layer (final device, 1× magnification). (**i**) Energy-dispersive

X-ray spectroscopy (EDX) spectrum after complete processing of the wafer to confirm the absence of relevant surface contaminants.

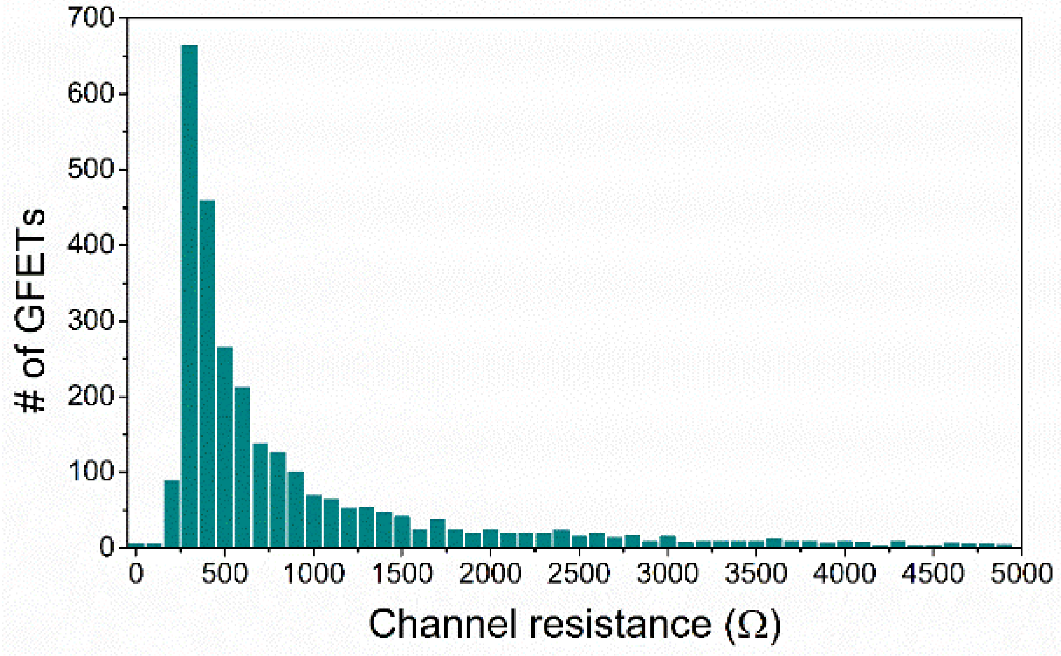

(**a**)

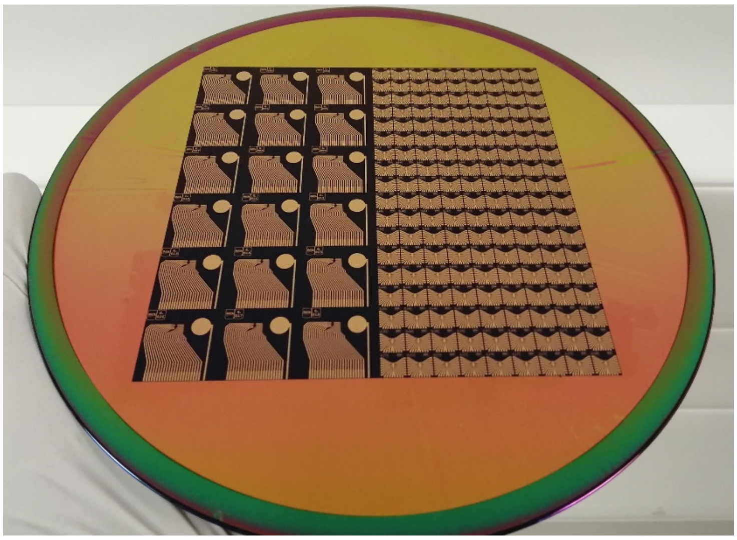

(**b**)

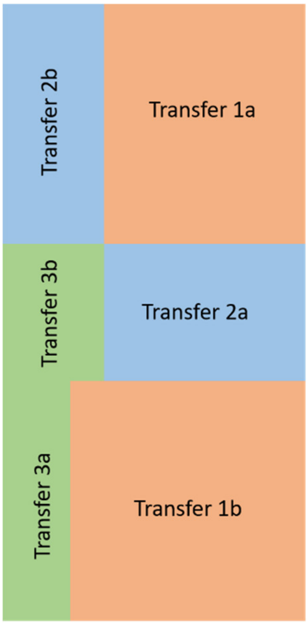

(**c**)

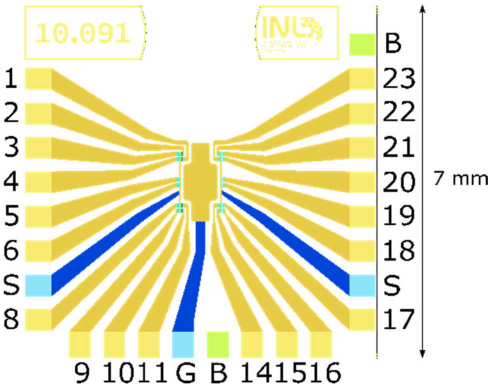

(**d**)

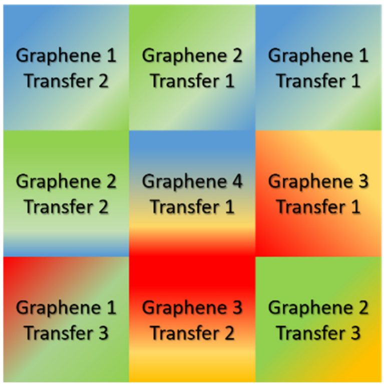

(**e**)

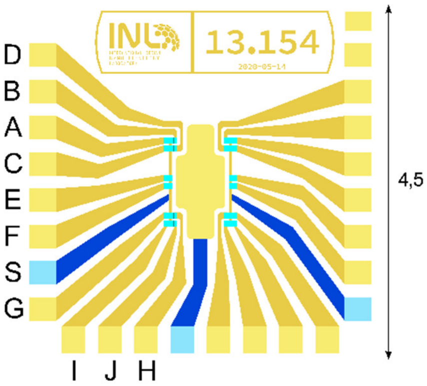

(**f**)

Figure S15. Complementary information of the electrical characterization of wafers A and B. (**a**) Histogram of wafer A GFET channel resistances up to 5 kΩ (87% of measured devices). (**b**) Photograph of wafer A showing two different designs. Only the right-half of wafer A is used in the statistical analysis, since it refers to the design optimized during this paper. (**c**) Distribution of graphene patches over wafer A from a single chemical vapor deposition (CVD) batch. (**d**) Layout of one die from wafer A with the map of contacts used in Figure 15a of the main text. (**e**) Distribution of graphene patches over wafer B from CVD batches 1 to 4. Graphene from batches 1 and 2 was divided in three pieces, while that from batch 3 was divided into two pieces, and a single piece was used from batch 4. (**f**) Layout of one die from wafer B with the map of contacts used in Figure 15b of the main text. Note that not all the contacts were used in this study.
